# Efficacy of Follicle-Stimulating Hormone (FSH) Alone, FSH + Luteinizing Hormone, Human Menopausal Gonadotropin or FSH + Human Chorionic Gonadotropin on Assisted Reproductive Technology Outcomes in the “Personalized” Medicine Era: A Meta-analysis

**DOI:** 10.3389/fendo.2017.00114

**Published:** 2017-06-01

**Authors:** Daniele Santi, Livio Casarini, Carlo Alviggi, Manuela Simoni

**Affiliations:** ^1^Unit of Endocrinology, Department of Biomedical, Metabolic and Neural Sciences, University of Modena and Reggio Emilia, Modena, Italy; ^2^Unit of Endocrinology, Department of Medicine, Endocrinology, Metabolism and Geriatrics, Azienda OU of Modena, Modena, Italy; ^3^Center for Genomic Research, University of Modena and Reggio Emilia, Modena, Italy; ^4^Department of Neuroscience, Reproductive Science and Odontostomatology, University of Naples Federico II, Napoli, Italy

**Keywords:** follicle-stimulating hormone, luteinizing hormone, human chorionic gonadotropin, human menopausal gonadotropin, pregnancy rate, assisted reproductive technology, controlled ovarian stimulation

## Abstract

**Setting:**

Luteinizing hormone (LH) and human chorionic gonadotropin (hCG) act on the same receptor, activating different signal transduction pathways. The role of LH or hCG addition to follicle-stimulating hormone (FSH) as well as menopausal gonadotropins (human menopausal gonadotropin; hMG) in controlled ovarian stimulation (COS) is debated.

**Objective:**

To compare FSH + LH, or FSH + hCG or hMG vs. FSH alone on COS outcomes.

**Design:**

A meta-analysis according to PRISMA statement and Cochrane Collaboration was performed, including prospective, controlled clinical trials published until July 2016, enrolling women treated with FSH alone or combined with other gonadotropins. Trials enrolling women with polycystic ovarian syndrome were excluded (PROSPERO registration no. CRD42016048404).

**Results:**

Considering 70 studies, the administration of FSH alone resulted in higher number of oocytes retrieved than FSH + LH or hMG. The MII oocytes number did not change when FSH alone was compared to FSH + LH, FSH + hCG, or hMG. Embryo number and implantation rate were higher when hMG was used instead of FSH alone. Pregnancy rate was significantly higher in FSH + LH-treated group vs. others. Only 12 studies reported live birth rate, not providing protocol-dependent differences. Patients’ stratification by GnRH agonist/antagonist identified patient subgroups benefiting from specific drug combinations.

**Conclusion:**

In COS, FSH alone results in higher oocyte number. HMG improves the collection of mature oocytes, embryos, and increases implantation rate. On the other hand, LH addition leads to higher pregnancy rate. This study supports the concept of a different clinical action of gonadotropins in COS, reflecting previous *in vitro* data.

## Introduction

Luteinizing hormone (LH) and human chorionic gonadotropin (hCG) are heterodimeric glycoprotein hormones, acting on the same receptor (LHCGR) (1). These gonadotropins were considered equivalent at the molecular level for long time, until the demonstration of specific intracellular-mediated signaling (2). *In vitro* models of human granulosa cells demonstrated that hCG is more potent than LH in inducing cyclic adenosine monophosphate production (cAMP) production (2), while the latter leads to preferential ERK1/2 and AKT pathways activation (2). Thus, although LH and hCG activate different kinetics (2, 3), whether and how they differently influence *in vivo* response remains unclear (4).

In humans, follicle-stimulating hormone (FSH) and LH act in concert to stimulate folliculogenesis and ovulation. Therefore, these gonadotropins are used in the controlled ovarian stimulation (COS) in order to produce relatively high oocyte number to be used fresh or after cryopreservation (5) to obtain pregnancies. The physician identifies the presumably most appropriate regimen, in terms of gonadotropin-releasing hormone (GnRH) analog protocol, FSH formulation, starting FSH dose, and combination of different gonadotropins, following the evaluation of demographic, anthropometric, and ovarian reserve profiles (6–8). Generally, FSH is selected as standard treatment, and hCG or LH may be added. The knowledge of human physiology provides a rationale for LH activity supplementation during COS. Although *in vitro* and animal models provided the evidences of hormone-specific actions, the choice of the optimal gonadotropin combination to be used in COS is not well standardized and remains entrusted to clinician’s decision. Especially, the pregnancy hormone hCG is generally used to obtain LH-like activity and support of multi-follicle growth since decades (9). With this in mind, human menopausal gonadotropin (hMG) is commonly used as preparation with LH-like activity, due to the presence of LH and/or hCG molecules. hMG alone and hCG/LH + FSH were repeatedly proposed (10, 11) but some unfavorable results, in particular in terms of number of oocytes retrieved (12, 13), provided concerns about the usefulness of addition of “LH activity.”

Currently, the gonadotropin market offers a wide choice, including urinary and recombinant preparations of FSH, LH, hCG, and hMG alone or in various combinations, recently further enriched by biosimilars. This palette of competitor drugs, registered for the same indication but biochemically and physiologically different, introduced the concept of “personalized” assisted reproductive technology (ART) schemes, which is very attractive for patients and doctors but not supported by solid evidence and largely industry-promoted. These gonadotropins show different kinetics in *in vitro* models, but no clear *in vivo* differences in COS are available so far. Most studies have been tried to answer the question of what is the best gonadotropin combinations, although inconclusive results were achieved, not sufficient to guide a really evidence-based, personalized choice in ART. Indeed, no powerful, properly designed, controlled prospective clinical trials are available to support the rationale of any COS scheme so far. As a matter of fact, the design of randomized clinical trials is challenging in this setting, due to the peculiar emotional situation and heterogeneity of the infertile population together with the time and costs required. Thus, 64 meta-analyses have been published to compare different ART approaches and outcomes (Table 1). However, each review is focused on a specific single comparison (e.g., hMG vs. FSH, GnRH agonist vs. antagonists, etc.) in a peculiar clinical setting. In particular, 25 systematic reviews compared the efficacy of different GnRH analogs, 17 compared urinary and recombinant FSH preparations, and only 6 evaluated the efficacy of LH supplementation to FSH (Table 1). None of these comparisons provided a comprehensive analysis of entire process, from oocyte recruitment to live birth rate, and their conclusions are rarely translated in clinical practice. In fact, no accepted guideline exists in this field of medicine in which registered indications and reimbursability of gonadotropins by the national health care systems are guided by costs rather than scientific evidence/clinical outcome.

**Table 1:** **Previous meta-analysis characteristics**.

First author	Journal	Year	Comparison	End-points	Number of studies
Daya	Fertil Steril	1995	U-follicle-stimulating hormone (FSH) vs. r-FSH	Pregnancy rate	8
Daya	Cochrane Database Syst Rev	1996	U-FSH vs. r-FSH		Withdrawan
Daya	Hum Reprod	1999	U-FSH vs. r-FSH	Oocytes retrieved	12
Nugent	Cochrane Database Syst Rev	2000	Different u-FSH in polycystic ovarian syndrome (PCOS)	Pregnancy rate	23
Daya	Cochrane Database Syst Rev	2000	U-FSH vs. r-FSH	Pregnancy rate	18
van Wely	Fertil Steril	2003	Human menopausal gonadotropin (hMG) vs. r-FSH	Pregnancy rate	6
Al-Inany	Hum Reprod	2003	U-FSH vs. r-FSH	Oocytes retrieved	20
Albuquerque	Cochrane Database Syst Rev	2005	Depot gonadotropin-releasing hormone (GnRH) agonist vs. daily GnRH agonist	Pregnancy rate	6
Pandian	Cochrane Database Syst Rev	2005	*In vitro* fertilization (IVF) vs. intrauterine insemination (IUI)	Pregnancy rate	10
Sallam	Cochrane Database Syst Rev	2006	GnRH agonist timing in endometriosis	Pregnancy rate	3
Griesinger	Reprod Biomed Online	2006	GnRH agonist vs. GnRH antagonist in PCOS	Oocytes retrieved	13
Franco	Reprod Biomed Online	2006	GnRH agonist vs. GnRH antagonist in PCOS	Oocytes retrieved	6
Sunkara	Reprod Biomed Online	2007	GnRH agonist vs. GnRH antagonist	Oocytes retrieved	9
Mochtar	Cochrane Database Syst Rev	2007	R-luteinizing hormone (LH) plus r-FSH vs. r-FSH	Live birth rate	14
Pandian	Cochrane Database Syst Rev	2007	Different GnRH analog protocols	Live birth rate	9
Daya	Cochrane Database Syst Rev	2007	U-FSH vs. r-FSH		Withdrawan
Kolibianakis	Hum Reprod Update	2007	R-LH plus r-FSH vs. r-FSH in GnRH antagonist	Live birth rate	5
Baruffi	Reprod Biomed Online	2007	R-LH plus r-FSH vs. r-FSH in GnRH antagonist	Oocytes retrieved	5
Al-Inany	Reprod Biomed Online	2008	hMG vs. r-FSH	Live birth rate	10
Coomarasamy	Hum Reprod	2008	U-FSH vs. r-FSH	Live birth rate	7
Al-Inany	Reprod Biomed Online	2008	hMG vs. r-FSH	Live birth rate	5
Al-Inany	Gynecol Endocinol	2009	hMG vs. r-FSH	Pregnancy rate	6
Jee	Gynecol Obstet Invest	2010	hMG vs. r-FSH	Pregnancy rate	10
Lehert	Reprod Biol Endocrinol	2010	hMG vs. r-FSH	Oocytes retrieved	16
Pandian	Cochrane Database Syst Rev	2010	GnRH agonist vs. GnRH antagonist	Live birth rate	15
Pandian	Cochrane Database Syst Rev	2010	Different GnRH analog protocols	Live birth rate	10
Sterrenburg	Hum Reprod Update	2011	Different r-FSH doses	Pregnancy rate	10
Al-Inany	Cochrane Database Syst Rev	2011	GnRH agonist vs. GnRH antagonist	Live birth rate	45
Youssef	Cochrane Database Syst Rev	2011	GnRH agonist vs. hCG for trigger	Live birth rate	11
van Wely	Cochrane Database Syst Rev	2011	hMG vs. r-FSH	Live birth rate	42
Youssef	Cochrane Database Syst Rev	2011	U-hCG vs. r-hCG	Live birth rate	14
Siristatidis	Cochrane Database Syst Rev	2011	Different GnRH agonist protocols	Pregnancy rate	29
Maheshwari	Cochrane Database Syst Rev	2011	Short vs. ultra-short GnRH agonist protocols	Pregnancy rate	29
Pundir	Hum Reprod	2011	GnRH agonist vs. GnRH antagonist	Oocytes retrieved	14
Bodri	Fertil Steril	2011	GnRH agonist vs. GnRH antagonist	Pregnancy rate	8
van Wely	Hum Reprod Update	2012	hMG vs. r-FSH	Live birth rate	42
Hill	Fertil Steril	2012	R-LH plus r-FSH vs. r-FSH in GnRH antagonist	Pregnancy rate	7
Konig	Fertil Steril	2012	R-LH plus r-FSH vs. r-FSH in GnRH antagonist in women older than 35 years	Pregnancy rate	9
Mahmoud Youssef	Fertil Steril	2012	Long acting FSH vs. r-FSH	Pregnancy rate	4
Pandian	Cochrane Database Syst Rev	2012	IVF vs. IUI	Pregnancy rate	6
Gibreel	Cochrane Database Syst Rev	2012	Gonadotropins vs. clomiphene citrate	Live birth rate	14
Pouwer	Cochrane Database Syst Rev	2012	Long acting FSH vs. r-FSH	Live birth rate	4
Pundir	Reprod Biomed Online	2012	GnRH agonist vs. GnRH antagonist in PCOS	OHSS rate	9
Albuquerque	Cochrane Database Syst Rev	2013	Depot GnRH agonist vs. daily GnRH agonist	Pregnancy rate	16
Matsaseng	Gynecol Obstet Invest	2013	Mild ovarian stimulations vs. traditional IVF	Pregnancy rate	5
Xiao	Fertil Steril	2013	GnRH agonist vs. GnRH antagonist	Pregnancy rate	12
Fan	Gynecol Endocinol	2013	rLH supplementation in poor responders	Pregnancy rate	3
Xiao	Gynecol Endocinol	2013	GnRH agonist vs. GnRH antagonist	Oocytes retrieved	7
Youssef	Cochrane Database Syst Rev	2014	GnRH agonist vs. hCG for trigger	Live birth rate	17
Xiao	PlosONE	2014	GnRH agonist vs. GnRH antagonist	Oocytes retrieved	23
Chen	Gynecol Endocinol	2014	Timing of hCG administration	Oocytes retrieved	7
Lin	PlosONE	2014	GnRH agonist vs. GnRH antagonist	Pregnancy rate	9
Hu	J Int Med Res	2014	LH priming vs. FSH alone	Estradiol serum levels	3
Song	Gynecol Endocinol	2014	GnRH agonist vs. letrozole	Pregnancy rate	3
Siristatidis	Cochrane Database Syst Rev	2015	different GnRH agonist protocols	Pregnancy rate	37
Weiss	Cochrane Database Syst Rev	2015	U-FSH vs. r-FSH in PCOS	Live birth rate	14
Nugent	Cochrane Database Syst Rev	2015	Different u-FSH in PCOS		Withdrawan
Nahuis	Cochrane Database Syst Rev	2015	U-FSH vs. r-FSH in PCOS		Withdrawan
Pandian	Cochrane Database Syst Rev	2015	IVF vs. IUI	Pregnancy rate	8
Pouwer	Cochrane Database Syst Rev	2015	Long acting FSH vs. r-FSH	Live birth rate	6
Youssef	J Adv Res	2015	GnRH agonist vs. hCG for trigger	Pregnancy rate	19
Fensore	J Ovar Res	2015	Long acting FSH vs. r-FSH	Oocytes retrieved	7
Al-Inany	Cochrane Database Syst Rev	2016	GnRH agonist vs. GnRH antagonist	Live birth rate	63
Youssef	Cochrane Database Syst Rev	2016	U-hCG vs. r-hCG	Live birth rate	18

Having in mind physiology and the different *in vitro* effects of LH and hCG, in this work, we addressed the question whether LH, LH-like activity, and hCG could have different results on COS outcomes. To this purpose, we evaluated the efficacy of LH or hCG plus FSH or hMG alone, compared to what is considered the standard care for COS, i.e., the use of FSH alone, using a meta-analytic approach. This is the first meta-analysis in which all gonadotropin combinations are considered. Moreover, a full-spectrum evaluation of all ART endpoints is provided, to recognize when and how LH, LH-activity, and hCG influence ART outcomes.

## Materials and Methods

We performed a meta-analysis according to the Cochrane Collaboration and PRISMA statement. The meta-analysis was accepted in the International Prospective Register of Systematic Reviews (PROSPERO; registration n. CRD42016048404) prior to commencing the study, ensuring transparency and originality of the review process.

### Data Sources and Searches

We conducted a comprehensive literature search for English-language articles in MEDLINE (PubMed), EMBASE, Cochrane Library, SCOPUS, and UpToDate, published until July 2016. Search key words were as follows: controlled ovarian stimulation (COS), controlled ovarian hyperstimulation (COH), ART, *in vitro* fertilization (IVF), intracytoplasmatic sperm injection (ICSI), luteinizing hormone (LH), follicle stimulating hormone (FSH), human menopausal gonadotropin (hMG), hCG, follitropin, oocytes retrieved, and pregnancy. The Boolean functions AND and OR were used to combine key words listed above.

### Study Selection and Inclusion Criteria

#### Types of Studies

The inclusion criteria, established before the literature search, were

- Prospective, longitudinal, and controlled clinical trials;
- Enrollment of women without limits of age;
- Treatment with LH or hCG or hMG during the follicular development phase.

Retrospective studies were not included. Similarly, trials enrolling women with polycystic ovarian syndrome (PCOS) were excluded, due to peculiar endocrine features of these patients. The ART methodology chosen was not an inclusion or exclusion criterion. However, each outcome was further evaluated considering the studies on the basis of the ART protocol used. Finally, randomization was not considered a strict inclusion criterion, thus randomized, semirandomized, and non-randomized clinical trials were reviewed. Therefore, all available controlled studies were considered increasing sample size, in spite of the wide range of clinical protocols available.

#### Type of Participants

Women undergoing COS for ART were considered. No inclusion criteria were applied for the male partner of the infertile couple.

#### Type of Interventions

All ART stimulation protocols were considered and studies included provided the comparison between LH, hCG, or hMG in the follicular phase with FSH.

### Data Collection Process and Quality

Two authors (Santi Daniele and Casarini Livio) extracted the abstracts from all studies found through literature search until July 2016. All abstracts were evaluated for inclusion criteria, and data were extracted from each study considered eligible, with regard to study design, year of publication, number of included/excluded subjects, number of dropped-out patients, and the use of intention to treat or per protocol analysis.

The quality of trials was assessed using the parameters proposed by Jadad et al. (14) and Table 2 summarizes the features of the selected studies.

**Table 2:** **Characteristics of included studies**.

				Control group									Study group								

Authors	Year	Protocol used	ART	Number	Mean age (years)	Drug 1	Name	Startig doe (IU/daily)	Drug 2	name	Startig doe (IU/daily)	Drop out	Numr	Mean age (years)	Drug 1	name	Startig doe (IU/daily)	Drug 2	name	Startig doe (IU/daily)	Drop out
Gerli	1993	Gonadotropin-releasing hormone (GnRH) agonist	*In vitro* fertilization (IVF)	17	30.9	FSH	Metrodin	225				2	15	31.4	hMG	Pergonal	225				1
Daya	1995	GnRH agonist	IVF	115	33.5	FSH	Metrodin	150					117	33.2	hMG	Pergonal	150				
Westergaard	1996	GnRH agonist	IVF	104	31.0	FSH	Fertinorm	225					114	32.0	hMG	Pergonal	225				
Jansen	1998	None	IVF	47	32.0	FSH	Puregon	150					32	31.1	hMG	Humegon	225				
Filicori	1999	GnRH agonist	IVF	10	32.0	FSH	Metrodin	300				0	10	33.0	FSH	Metrodin	300	hCG	Profasi	50	0
Sills	1999	GnRH agonist	IVF	17	35.4	FSH	Fertinex						14	36.7	FSH	Fertinex		LH	Lhadi	75	
Balasch	2001	GnRH agonist	IVF	14	33.6	FSH	Gonal F	150				1	16	34.8	FSH	Gonal F	150	LH	Luveris	75	1
De Placido	2001	GnRH agonist	IVF	40	30.1	FSH	Gonal F	300				0	20	31.6	FSH	Gonal F	150	hMG	Menogon	150	0
Filicori	2001	GnRH agonist	IVF	25	32.0	FSH	Metrodin	150				0	25	33.0	hMG	Menogon	150				0
Gordon	2001	GnRH agonist	IVF	69	33.5	FSH	Puregon	225				12	59	33.5	hMG	Humegon	75				6
Ng	2001	GnRH agonist	IVF	20	33.5	FSH	Gonal F	300					20	32.0	hMG	Pergonal	300				
Strehler	2001	GnRH antagonist	IVF	248	32.3	FSH	Gonal F	300					259	31.8	hMG	Menogon	300				
Westergaard	2001	GnRH agonist	IVF	190		FSH	Gonal F	225				2	189		hMG	Menogon	225				3
Filicori	2002	GnRH agonist	IVF	30	31.9	FSH	Metrodin	150					90	32.7	FSH	Metrodin	150	LH	Menogon	75	
Ismail	2002	GnRH agonist	IVF	75	33.2	FSH	Fostimon	150					78	34.3	hMG	Menogon	150				
Lisi	2002	GnRH agonist	IVF	331	34.7	FSH	Gonal F	150					122	34.8	FSH	Gonal F	150	LH	Luveris	75	
Filicori a	2003	GnRH agonist	Intrauterine insemination (IUI)	25	31.9	FSH	Gonal F	150					25	32.6	hMG	Menogon	150				
Filicori b	2003	GnRH agonist	IVF	50	25.9	FSH	Gonal F	150				14	50	27	hMG	Menopur	150				12
Ku	2003	GnRH agonist	IVF	19	34.6	FSH	Metrodin	300					26	33.0	FSH	Metrodin	300	hMG	Pergonal	75	
Marrs	2003	GnRH agonist	IVF	219	31.9	FSH	Gonal F	225					212	32.4	FSH	Gonal F	225	LH	Luveris	150	
Acevedo	2004	GnRH antagonist	IVF	20	23.0	FSH	Gonal F	225					22	26.0	FSH	Gonal F	225	LH	Luveris	75	
Cédrin-Durnerin	2004	GnRH antagonist	IVF	96	31.7	FSH	Gonal F	150				2	107	31.4	FSH	Gonal F	150	LH	Luveris	75	0
De Placido	2004	GnRH agonist	IVF	46	30.4	FSH	Gonal F	150					46	30.0	FSH	Gonal F	150	LH	Luveris	75	
Ferraretti	2004	GnRH agonist	IVF	104	31.7	FSH	Gonal F	225				2	54	31.5	FSH	Gonal F	225	LH	Luveris	75	4
Ferraretti	2004	GnRH agonist	IVF	104	31.7	FSH	Gonal F	225				2	22	32.0	FSH	Gonal F	225	hMG	Menogon		
Humaidan	2004	GnRH agonist	IVF	115	30.5	FSH	Puregon	150					116	30.8	FSH	Puregon	150	LH	Luveris		
Loutradis	2004	GnRH agonist	IVF	106	37.3	FSH		200					98	38.1	FSH		200	hMG			
De Placido	2005	GnRH agonist	IVF	58	30.4	FSH	Gonal F	225					57	31.5	FSH	Gonal F	225	LH	Luveris	150	
Drakakis	2005	GnRH agonist	IVF	22	33.0	FSH	Puregon	200					24	32.4	FSH	Puregon	200	hMG	Menogon	75	
Filicori	2005	GnRH agonist	IVF	24	33.4	FSH	Puregon	225					24	33.8	FSH	Puregon	225	hCG	Gonasi	200	
Gómez-Palomares	2005	GnRH antagonist	IVF	58	39.0	FSH	Gonal F	225	hMG	HMG-Lepori	75	4	36	38.8	FSH	Gonal F	300	LH	Luveris	75	2
Griesinger	2005	GnRH antagonist	IVF	65	30.5	FSH	Gonal F	150				11	62	30.3	FSH	Gonal F	150	LH	Luveris	75	6
Hugues	2005	None	IVF	30	29.9	FSH	Gonal F	150				0	117	29.3	FSH	Gonal F	150	LH	Luveris	150–300	1
Fabregues	2006	GnRH agonist	IVF	60	38.2	FSH	Gonal F	150				5	60	38.4	FSH	Gonal F	150	LH	Luveris	150	5
Levi-Setti	2006	GnRH antagonist	IVF	20	32.3	FSH	Gonal F	225				4	20	32.2	FSH	Gonal F	150	LH	Luveris	75	2
Tarlatzis	2006	GnRH agonist	IVF	59	30.3	FSH	Gonal F	150				2	55	30.5	FSH	Gonal F	150	LH	Luveris	75	0
Berkkanoglu	2007	GnRH agonist	IVF	51	34.9	FSH	Gonal F	600					46	36.3	FSH	Gonal F	600	LH	Luveris	75	
Berkkanoglu	2007	GnRH agonist	IVF	51	34.9	FSH	Gonal F	600					48	35.2	FSH	Gonal F	600	hCG	Ovitrelle	75	
Demirol	2007	None	IUI	161	30.4	FSH	Gonal F	150				0	80	30.8	hMG		150				0
Ziebe	2007	GnRH agonist	IVF	368		FSH		225					363		hMG		225				
Barrenetxea	2008	GnRH agonist	IVF	42	41.8	FSH	Gonal F	300					42	42.1	FSH	Gonal F	300	LH	Luveris	150	
Bosch	2008	GnRH antagonist	IVF	140	33.4	FSH	Gonal F	225				20	140	33.2	hMG	Menopur	225				23
Hompes	2008	GnRH antagonist	IVF	317	32.0	FSH	Gonal F	150				15	312	31.7	hMG	Menopur	150				19
Nyboeandersen	2008	GnRH agonist	IVF	261	31.8	FSH	Gonal F	150				0	265	31.7	FSH	Gonal F	150	LH	Luveris	75	0
Blockeel	2009	GnRH antagonist	IVF	35	30.0	FSH	Puregon	225				3	35	29.0	FSH	Puregon	225	hCG	Pregnyl	200	6
Check	2009	GnRH antagonist	IVF	35	35.1	FSH		300				1	35	33.6	FSH		300	hCG		25	3
Drakakis	2009	GnRH agonist	IVF	58	36.4	FSH	Gonal F	200	rhCG		200		56	37.3	FSH	Gonal F	200	LH			
Matorras	2009	GnRH agonist	IVF	68	36.7	FSH	Gonal F	300				3	63	36.6	FSH	Gonal F	300	LH	Luveris	150	0
Melo	2010	GnRH agonist	IVF	346	24.9	FSH	Gonal F	225					333	23.9	hMG	Menopur	225				
Pacchiarotti	2010	GnRH antagonist	IVF	60		hMG	Menopur	225				2	62		FSH	Pergoveris	225	LH	Luveris		8
Bosch	2011	GnRH antagonist	IVF	314	34.6	FSH	Gonal F	225				50	311	34.7	FSH	Gonal F	150	LH	Luveris	75	56
Caserta	2011	GnRH agonist	IVF	501	34.8	FSH	Gonal F	150					498	34.3	FSH	Gonal F	150	LH			
Kokac	2011	GnRH agonist	IUI	24	29.5	FSH	Gonal F	75					25	28.8	hMG	Merional	75				
Pezzuto	2011	GnRH agonist	IVF	40	34.0	FSH	Puregon	225					40	35.0	FSH	Puregon	225	LH	Luveris	75	
Sagnella	2011		IUI	262	35.4	FSH	Gonal F	150				23	261	35.0	hMG	Meropur	75–150				5
Barberi	2012	GnRH agonist	IVF	11	32.3	FSH	Gonal F	150				10	9	34.1	FSH	Gonal F	150	LH	Luveris	75	2
Devroy	2012	GnRH antagonist	IVF	375	30.4	FSH	Puregon	150				59	374	30.8	hMG	Menopur	150				69
Lisi	2012	GnRH agonist	IVF	75	32.8	FSH	Gonal F	150					75	33.6	FSH	Gonal F	150	LH		75	
Madani	2012	GnRH antagonist	IVF	26	39.2	FSH	Gonal F	300				0	47	38.9	FSH	Gonal F	300	hCG	Pregnyl	200	0
Revelli	2012	GnRH antagonist	IVF	266	39.2	FSH	Gonal F	300				27	264	39.4	FSH	Gonal F	150	LH	Luveris	150	29
Thuesen	2012	GnRH agonist	IVF	16	31.5	FSH	Puregon	150				2	46	32.6	FSH	Puregon	150	hCG	Predalon	100	5
Ye	2012	GnRH agonist	IVF	64	36.2	FSH	Gonal F	225					63	36.2	hMG	Menopur	225				
Konig	2013	GnRH antagonist	IVF	128	37.9	FSH	Gonal F	225				17	125	38.0	FSH	Gonal F	225	LH	Luveris	150	14
Rashidi	2013		IUI	132	28.7	FSH	Gonal F	75				3	127	29.1	hMG	Menogon	75				1
Thuesen	2013	GnRH agonist	IVF	16	32.3	FSH	Puregon	150				0	46	32.3	FSH	Puregon	150	hCG	Predalon	100	0
Razi	2014	GnRH agonist	IVF	20	31.3	FSH	Gonal F	150				0	20	31.8	FSH	Gonal F	150	LH	Luveris	75	0
Behre	2015	GnRH agonist	IVF	99	37.6	FSH	Gonal F	300				1	103	37.4	FSH	Gonal F	300	LH	Luveris	150	2
Moro	2015	none	IUI	289	37.9	hMG	Meropur	150				5	290	38.4	FSH	Gonal F	150	LH	Luveris	150	13
Vuong	2015	GnRH antagonist	IVF	120	38.0	FSH	Gonal F	300				11	120	38.0	FSH	Gonal F	300	LH	Pergoveris	150	18
Yilmaz	2015	GnRH agonist	IVF	87	29.0	FSH	Puregon						50	30.3	FSH	Puregon		LH	Luveris	75	
Younis	2016	GnRH antagonist	IVF	30	38.6	FSH	Gonal F	300				6	32	38.9	FSH	Gonal F	300	LH	Luveris	150	5

Although studies considered in the meta-analysis used different endpoints, we performed an overall meta-analysis considering all studies evaluating at least pregnancy rate or number of oocytes retrieved.

The investigators (DS and LC), using Cochrane risk-of-bias algorithm, independently assessed the risk-of-bias for all trials. The following quality criteria and methodological details were evaluated for each trial included in the meta-analysis: (i) method of randomization, even if the randomization was not an inclusion criterion; (ii) concealment of allocation; (iii) presence or absence of blinding to treatment allocation; (iv) duration and type of treatment and follow-up phases; (v) number of participants recruited, analyzed, or lost to follow-up; (vi) timing of trial; (vii) whether an intention to treat analysis was done; (viii) whether a power calculation was done; (ix) source of funding; and (x) criteria for including participants and assessing outcomes.

### Summary Measures

The primary outcome was the number of oocytes retrieved, evaluated as mean difference between the two types of treatment compared. The choice of the primary endpoint derived from the consideration that the number of oocytes retrieved is the unique endpoint available in almost all trials in ART setting. Moreover, our meta-analysis aimed at comparing the efficacy *in vivo* of gonadotropin combinations, and the number of oocytes retrieved best described pathophysiologically the first step influenced by gonadotropin administration, i.e., follicular and oocyte development. The oocytes number remains the first measurable and reproducible parameter to describe gonadotropin action *in vivo*.

In clinical practice, the main ART outcome remains live birth rate. However, this parameter was not considered as primary endpoint in our meta-analysis, since it is influenced by a large number of unquantifiable biases and variables. Indeed, the vast majority of clinical trials dedicated to ART outcome do not report this parameter. In fact, the step following oocyte collection, i.e., embryo development, is strongly influenced by another important confounding factor, i.e., sperm quality, which is usually (and unexplainably) disregarded. Further, implantation rate follows embryo development and it is, in turn, affected by other factors, such as the endometrium thickness and activity, which are usually not controlled for. Continuing until pregnancy and live birth rate, each step is influenced by a number of factors, not immediately dependent on gonadotropins. Accordingly, the relationship between live birth rate and oocytes retrieved is suggested in the literature (15), but not universally accepted (16, 17). For these reasons, it is not possible to identify a unique endpoint to evaluate COS outcomes. Thus, we considered each available COS outcome after the number of oocytes retrieved as secondary endpoints, i.e., MII oocytes number, embryos, implantation rate, pregnancy rate, and live birth rate. Moreover, FSH dosage used and the ratio FSH dosage/number of oocytes retrieved were evaluated in order to describe the amount of gonadotropin needed to obtain each oocyte.”

### Data Synthesis and Analysis

The meta-analysis was conducted using the Review Manager (RevMan) software (Version 5.3.1 Copenhagen: The Nordic Cochrane Center, The Cochrane Collaboration, 2014). Data were combined using the fixed effect model and weighted mean differences, and 95% confidence intervals were estimated for each endpoint. The random effect model was used when high heterogeneity resulted among studies, as evaluated by *I*^2^ statistics. Meta-regression analyses were performed to evaluate the relationship between continuous variables.

Values of *p* < 0.05 were considered statistically significant.

### Risk-of-Bias across Studies

Two authors (Santi Daniele and Casarini Livio) independently evaluated risk-of-bias. Although randomization is not a strict inclusion criterion, it was evaluated as source of biases following the suggestions provided by the Cochrane collaboration.

## Results

**Figure 1:**
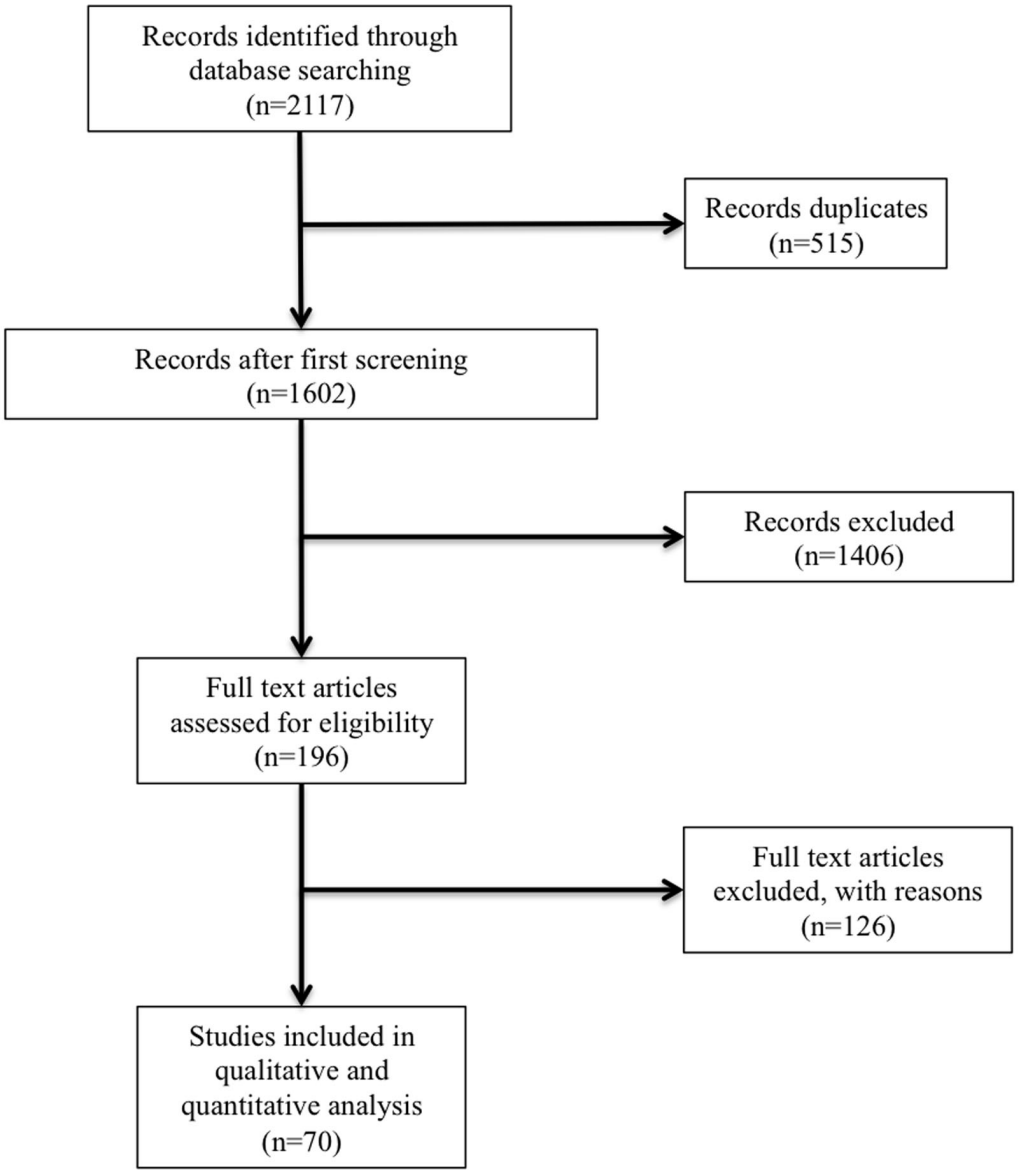
**Study flow chart**.

Of the 2,117 publications initially identified, 1,602 remained after duplicates removal. According to the strategy research, we identified 196 potentially relevant studies, based on the information given in the abstract. All trials were thoroughly appraised for eligibility in the meta-analysis and methodological quality. Seventy studies were included in the final analysis (Table 2; Figure 1).

### Considerations on Study Design

The mean age of all patients was 33.21 ± 3.43 years. Considering the wide heterogeneity in clinical trials included in the analysis, regarding inclusion criteria, FSH starting dose chosen and ART approaches, several subgroup analyses were performed (Table 3). In a subgroup analyses, studies were divided according to the GnRH analog used, agonist or antagonist, respectively. In subgroup analyses, three studies were excluded considering that hMG was administered together with FSH (18–20). An insufficient number of studies were available on the comparison between FSH alone vs. FSH + hCG and between FSH + LH vs. FSH + hCG, limiting the possibility to subgroup studies. Finally, considering the whole group of studies included in the meta-analysis, the ART approaches chosen after COS were different, ranging from intrauterine insemination (IUI) to intracytoplasmatic sperm injection (ICSI). However, only four studies evaluated IUI (21–24), thus the vast majority of trials included in the analysis considered IVF/ICSI. Moreover, of these four studies, three compared hMG to FSH alone (21–23) and one LH + FSH to FSH + hCG alone (24). Thus, a subgroup analysis, excluding studies performing IUI, was performed.

**Table 3:** **Number of studies evaluated in each comparison and in each subgroup analysis**.

	FSH + LH vs. FSH alone	FSH + hCG vs. FSH alone	hMG vs. FSH alone
Overall analyses	34	9	29
**Subgroup analyses**			
GnRH antagonists	10	3	5
GnRH agonists	22	6	20
GnRH analogs missing data	*2*	*0*	*4*
*In vitro* fertilization/intracytoplasmatic sperm injection	33	9	26
Intrauterine insemination	1	0	3
ART schemes missing information	*0*	*0*	*0*

*ART, assisted reproductive technology; FSH, follicle-stimulating hormone; GnRH, gonadotropin-releasing hormone; hCG, human chorionic gonadotropin; LH, luteinizing hormone*.

### Number of Oocytes Retrieved

**Figure 2:**
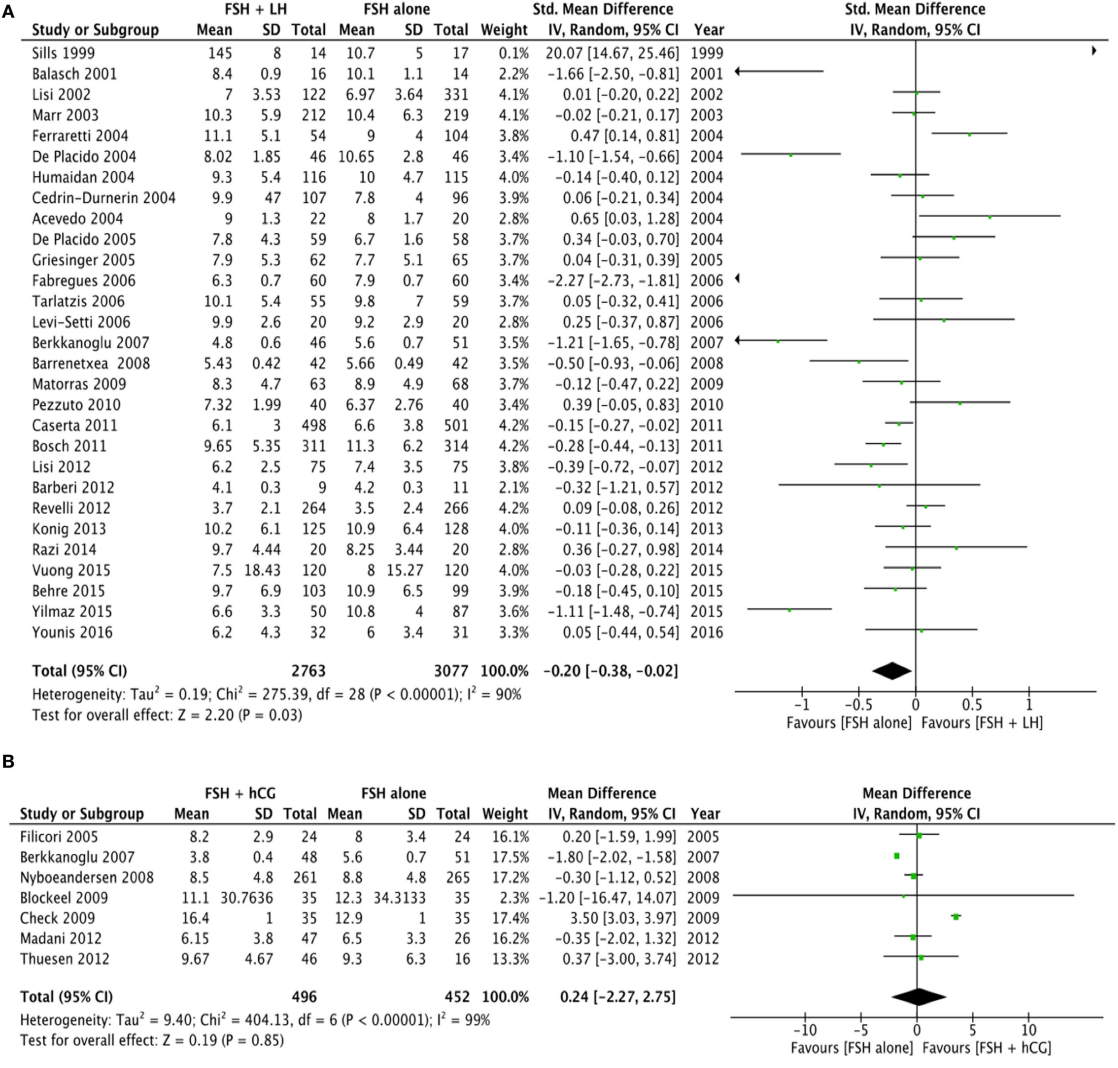

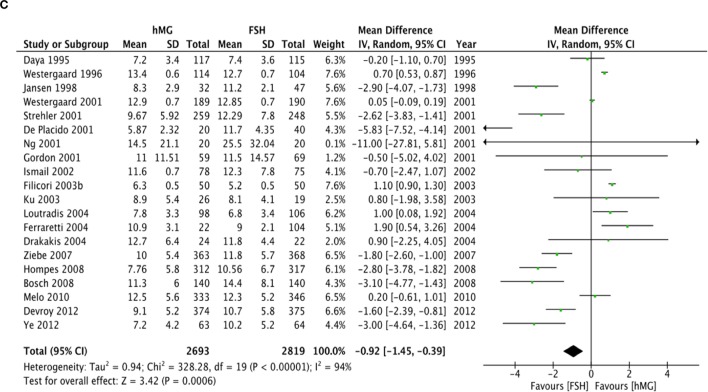
**Forrest plot evaluating the retrieved oocytes number comparing follicle-stimulating hormone alone to luteinizing hormone (A), human chorionic gonadotropin (B), and human menopausal gonadotropin (C)**.

Twenty-nine studies evaluated the comparison of FSH alone vs. FSH + LH, for a total of 5,840 patients. Studies using FSH alone retrieved a significantly higher number of oocytes compared to FSH + LH treatment (*p* = 0.010) (Figure 2A; Table 4). However, different results were found depending on COS protocol. In particular, higher oocyte numbers were retrieved when FSH was administered alone in a GnRH agonist protocol (*p* = 0.010), while no differences were observed in GnRH antagonist protocol (*p* = 0.840) (Table 4).

**Table 4:** **Main results of meta-analyses subgroups**.

	Luteinizing hormone (LH) + follicle-stimulating hormone (FSH) vs. FSH	Human chorionic gonadotropin (hCG) + FSH vs. FSH	Human menopausal gonadotropin (hMG) vs. FSH	LH + FSH vs. hCG + FSH
**Oocytes retrieved (mean difference)**				
Overall analysis	−0.20 (−0.36, −0.04)	0.24 (−2.27, 2.75)	−0.92 (−1.45, −0.39)	0.39 (−0.83, 1.61)
	***p* = 0.01**	*p* = 0.850	***p* < 0.001**	*p* = 0.530
	*I*^2^ = 88%	*I*^2^ = 99%	*I*^2^ = 94%	*I*^2^ = 96%
	
	29 studies	7 studies	20 studies	5 studies
	5,840 patients	948 patients	5,512 patients	538 patients

Gonadotropin-releasing hormone (GnRH) agonist	−0.35 (−0.63, −0.08)	–	−0.43 (−0.95, 0.10)	–
	***p* = 0.01**		*p* = 0.11	
	*I*^2^ = 93%		*I*^2^ = 93%	
	
	17 studies		16 studies	
	3,677 patients		3,347 patients	

GnRH antagonist	0.01 (−0.13, 0.16)	–	−2.38 (−3.10, −1.66)	–
	*p* = 0.840		***p* < 0.001**	
	*I*^2^ = 54%		*I*^2^ = 42%	
	
	10 studies		4 studies	
	2,163 patients		2,165 patients	

**FSH/oocytes (mean difference)**				
Overall analysis	−0.16 (−0.21, −0.11)	−0.04 (−0.17, 0.09)	0.17 (0.11, 0.23)	−0.25 (−0.94, 0.44)
	***p* < 0.001**	*p* = 0.550	***p* < 0.001**	*p* = 0.480
	*I*^2^ = 92%	*I*^2^ = 84%	*I*^2^ = 86%	*I*^2^ = 90%
	
	26 studies	6 studies	15 studies	4 studies
	5,404 patients	893 patients	4,436 patients	382 patients

GnRH agonist	−0.06 (−0.13, 0.01)	–	0.07 (−0.01, 0.14)	–
	*p* = 0.080		*p* = 0.090	
	*I*^2^ = 90%		*I*^2^ = 84%	
	
	18 studies		12 studies	
	3,613 patients		2,900 patients	

GnRH antagonist	−0.36 (−0.45, −0.26)	–	0.35 (0.25, 0.45)	–
	***p* < 0.001**		***p* < 0.001**	
	*I*^2^ = 95%		*I*^2^ = 74%	
	
	8 studies		3 studies	
	1,791 patients		1,536 patients	

**MII oocytes (mean difference)**				
Overall analysis	−0.27 (−0.56, 0.02)	−0.37 (−2.45, 1.71)	−0.60 (−1.31, 0.12)	−0.54 (−1.13, 0.05)
	*p* = 0.07	*p* = 0.730	*p* = 0.10	*p* = 0.07
	*I*^2^ = 94%	*I*^2^ = 91%	*I*^2^ = 89%	*I*^2^ = 92%
	
	20 studies	5 studies	11 studies	4 studies
	3,544 patients	352 patients	2,871 patients	424 patients

GnRH agonist	−0.50 (−1.01, 0.01)	–	0.15 (−1.30, 1.60)	–
	*p* = 0.05		*p* = 0.84	
	*I*^2^ = 96%		*I*^2^ = 86%	
	
	13 studies		7 studies	
	1,915 patients		706 patients	

GnRH antagonist	0.04 (−0.08, 0.15)	–	−1.36 (−1.51, −1.21)	–
	*p* = 0.54		***p* < 0.001**	
	*I*^2^ = 17%		*I*^2^ = 0%	
	
	7 studies		4 studies	
	1,629 patients		2,165 patients	

**Embryos (mean difference)**				
Overall analysis	−0.04 (−0.17, 0.10)	0.07 (−0.39, 0.53)	0.19 (0.07, 0.30)	−0.12 (−0.19, −0.06)
	*p* = 0.54	*p* = 0.77	***p* = 0.001**	***p* < 0.001**
	*I*^2^ = 83%	*I*^2^ = 74%	*I*^2^ = 94%	*I*^2^ = 83%
	
	26 studies	7 studies	16 studies	4 studies
	4,721 patients	918 patients	3,321 patients	500 patients

GnRH agonist	−0.07 (−0.25, 0.11)	–	0.23 (0.10, 0.35)	–
	*p* = 0.43		***p* < 0.001**	
	*I*^2^ = 88%		*I*^2^ = 95%	
	
	17 studies		13 studies	
	2,890 patients		2,589 patients	

GnRH antagonist	0.03 (−0.11, 0.18)	–	−0.02 (−0.19, 0.16)	–
	*p* = 0.64		*p* = 0.86	
	*I*^2^ = 36%		*I*^2^ = 74%	
	
	9 studies		3 studies	
	1,831 patients		732 patients	

**Implantation rate (mean difference)**				
Overall analysis	0.11 (0.00, 0.21)	−0.06 (−0.03, 0.01)	0.22 (0.02, 0.23)	−0.00 (−0.16, 0.15)
	*p* = 0.05	*p* = 0.59	***p* = 0.03**	*p* = 0.98
	*I*^2^ = 99%	*I*^2^ = 0%	*I*^2^ = 100%	*I*^2^ = 96%
	
	15 studies	5 studies	10 studies	4 studies
	2,669 patients	749 patients	3,208 patients	430 patients

GnRH agonist	0.16 (0.00, 0.31)	–	0.25 (−0.01, 0.51)	–
	*p* = 0.05		*p* = 0.06	
	*I*^2^ = 100%		*I*^2^ = 100%	
	
	10 studies		8 studies	
	1,256 patients		2,299 patients	

GnRH antagonist	0.01 (−0.08, 0.10)	–	0.15 (0.13, 0.17)	–
	*p* = 0.83		***p* < 0.001**	
	*I*^2^ = 85%		*I*^2^ = 0%	
	
	6 studies		2 studies	
	1,393 patients		909 patients	

**Pregnancy rate (odds ratio)**				
Overall analysis	1.20 (1.06, 1.37)	0.96 (0.72, 1.26)	1.10 (0.98, 1.22)	1.73 (1.26, 2.38)
	***p* = 0.004**	*p* = 0.750	*p* = 0.100	***p* < 0.001**
	*I*^2^ = 5%	*I*^2^ = 0%	*I*^2^ = 0%	*I*^2^ = 48%
	
	29 studies	8 studies	25 studies	5 studies
	5,665 patients	968 patients	6,894 patients	989 patients

GnRH agonist	1.27 (1.09, 1.48)	–	1.17 (1.01, 1.36)	–
	***p* = 0.002**		***p* = 0.030**	
	*I*^2^ = 9%		*I*^2^ = 0%	
	
	22 studies		17 studies	
	3,834 patients		3,627 patients	

GnRH antagonist	1.08 (0.87, 1.35)	–	1.10 (0.90, 1.34)	–
	*p* = 0.480		*p* = 0.370	
	*I*^2^ = 0%		*I*^2^ = 0%	
	
	9 studies		4 studies	
	1,831 patients		2,165 patients	

**Live birth rate (odds ratio)**				
Overall analysis	1.29 (0.91, 1.84)	–	1.13 (0.95, 1.33)	–
	*p* = 0.15		*p* = 0.17	
	*I*^2^ = 45%		*I*^2^ = 10%	
	
	5 studies	–	7 studies	–
	164 patients		747 patients	

*Bold character indicates significant results*.

Seven studies using FSH alone vs. FSH + hCG were compared, for a total of 948 patients. The overall analysis did not find significant differences in the number of oocytes retrieved between groups (*p* = 0.850) (Figure 2B; Table 4).

Twenty studies compared hMG with FSH for COS, for a total of 5,512 patients. Number of oocytes retrieved was significantly higher in FSH than hMG group (*p* < 0.001) (Figure 2C; Table 4). Four of these studies used a GnRH antagonist protocol, confirming the significant increase of oocytes retrieved (*p* < 0.001), but no difference was found in the 16 studies using GnRH agonist protocol (*p* = 0.110) (Table 4).

Finally, 5 studies evaluated the oocytes number comparing FSH plus LH to FSH plus hCG, for a total of 538 women. The analysis did not find significant difference between groups (*p* = 0.530) (Table 4).

### FSH Dose/Retrieved Oocyte Ratio

The FSH/retrieved oocyte ratio was significantly lower when LH was added to FSH (*p* < 0.001) (Table 4), as evaluated in 26 studies for a total of 5,404 women enrolled. However, different results were found considering the protocol of COS used. In particular, no significant difference was observed in GnRH agonist protocol (*p* = 0.080) (Table 4). On the contrary, a lower ratio was obtained when LH was added to FSH in the GnRH antagonist protocol (*p* < 0.001) (Table 4).

On the other hand, 6 studies compared the use of FSH alone with FSH plus hCG, for a total of 893 patients. The overall analysis did not find significant differences in the ratio between FSH dose and oocytes retrieved between groups (*p* = 0.550) (Table 4).

Fifteen studies compared hMG with FSH for COS, for a total of 4,436 patients. The ratio between FSH dose and the number of oocytes retrieved was significantly lower in the FSH compared to hMG group (*p* < 0.001) (Table 4). This significant difference was lost in the 12 studies using a GnRH agonist protocol (*p* = 0.090), while remained in the three studies using a GnRH antagonist protocol (*p* < 0.001) (Table 4).

Finally, 4 studies evaluated the ratio comparing FSH plus LH to FSH plus hCG, for a total of 382 women. No differences in the FSH/retrieved oocyte ratio were found between groups (*p* = 0.480) (Table 4).

### MII Oocytes

Twenty studies reported the MII oocytes number, comparing FSH alone and FSH + LH. The two groups did not differ considering the mean MII oocytes number (*p* = 0.050), even when GnRH agonist or antagonist protocols were considered separately (*p* = 0.050 and *p* = 0.540, respectively) (Table 4).

Five studies compared FSH alone vs. FSH + hCG, without finding differences in the mean MII oocytes number (*p* = 0.730) (Table 4).

Eleven studies compared FSH vs. hMG, finding no differences in the mean difference of MII oocytes (*p* = 0.100) (Table 4). Although this result remained also considering GnRH agonist protocols (*p* = 0.840), the MII oocytes number was significantly higher when FSH was used rather than hMG (*p* < 0.001) (Table 4).

Four studies compared directly FSH + LH vs. FSH + hMG, finding no difference in the MII oocytes number (*p* = 0.070) (Table 4).

### Embryos

Twenty-six studies reported the embryo number in the comparison between FSH alone vs. FSH + LH, without significant differences (*p* = 0.540) (Table 4). Similarly, no differences were observed in the GnRH agonist (*p* = 0.430) and antagonist group (*p* = 0.640).

Seven studies demonstrated a similar embryo number in the comparison of FSH alone vs. FSH + hCG (*p* = 0.770) (Table 4).

Sixteen studies described the embryo number in the comparison between FSH and hMG. In this subgroup, hMG showed a higher embryo number (*p* = 0.001), maintained when GnRH agonist was used (*p* < 0.001), but not in the GnRH antagonist group (*p* = 0.860) (Table 4).

The direct comparison between FSH + LH and FSH + hMG demonstrated a higher embryo number when FSH was used combined to LH (*p* < 0.001) (Table 4).

### Implantation Rate

The implantation rate was calculated as the ratio between number of gestational sacs and the number of transferred embryos. This was reported in 15 studies comparing FSH alone vs. FSH + LH, demonstrating a similar rate (*p* = 0.050), maintained both in GnRH agonist (*p* = 0.050) and antagonist protocols (*p* = 0.830) (Table 4).

Five studies demonstrated an equal implantation rate in the comparison FSH alone vs. FSH + hCG (*p* = 0.590) (Table 4).

Ten studies showed a higher implantation rate when hMG was used instead of FSH (*p* = 0.030) (Table 4). This result remained in the GnRH antagonist group (*p* < 0.001), but not in the GnRH agonist group (*p* = 0.060) (Table 4).

No different implantation rate was found when FSH + LH was directly compared to FSH + hMG (*p* = 0.980) (Table 4).

### Pregnancy Rate

**Figure 3:**
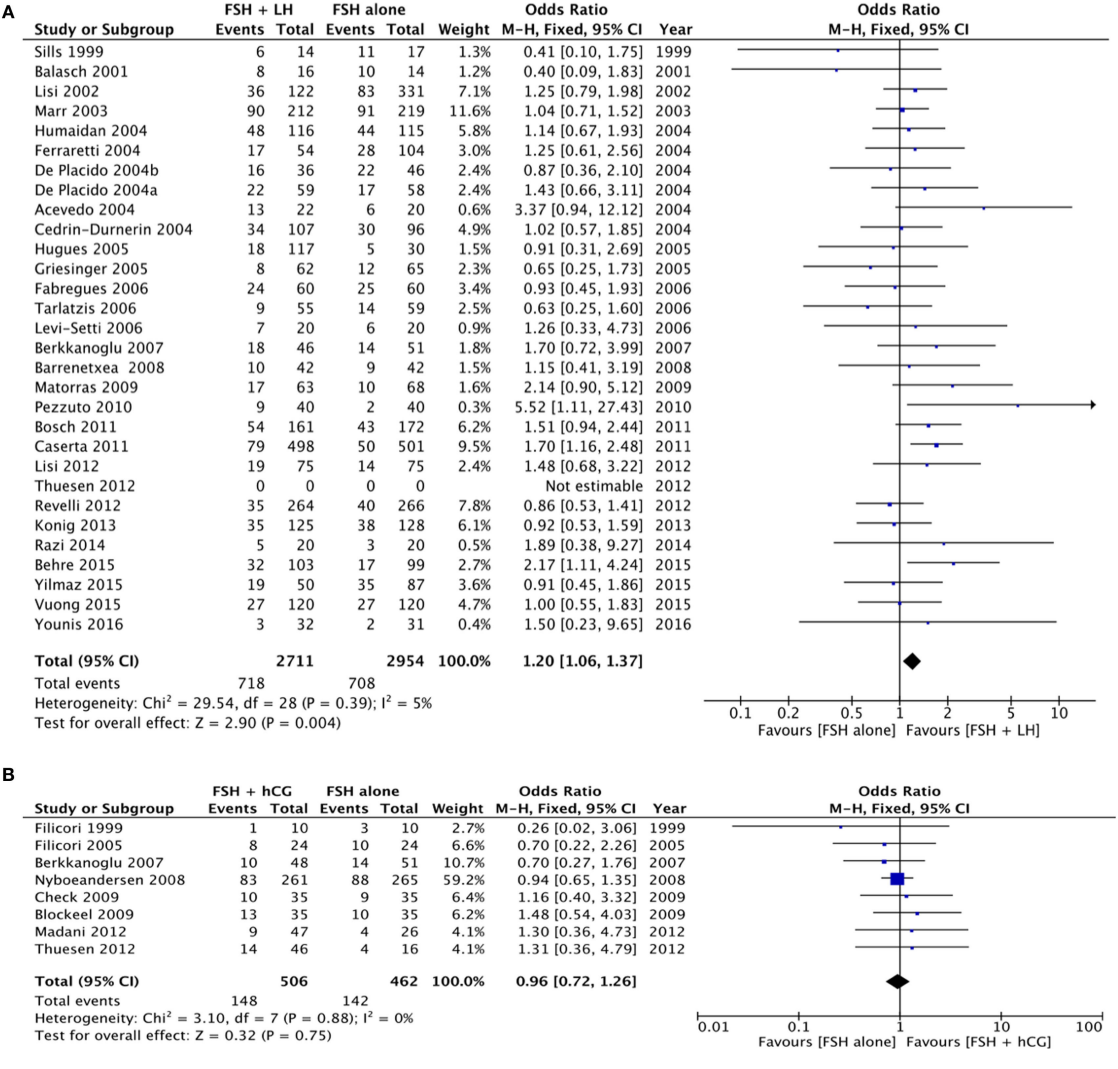

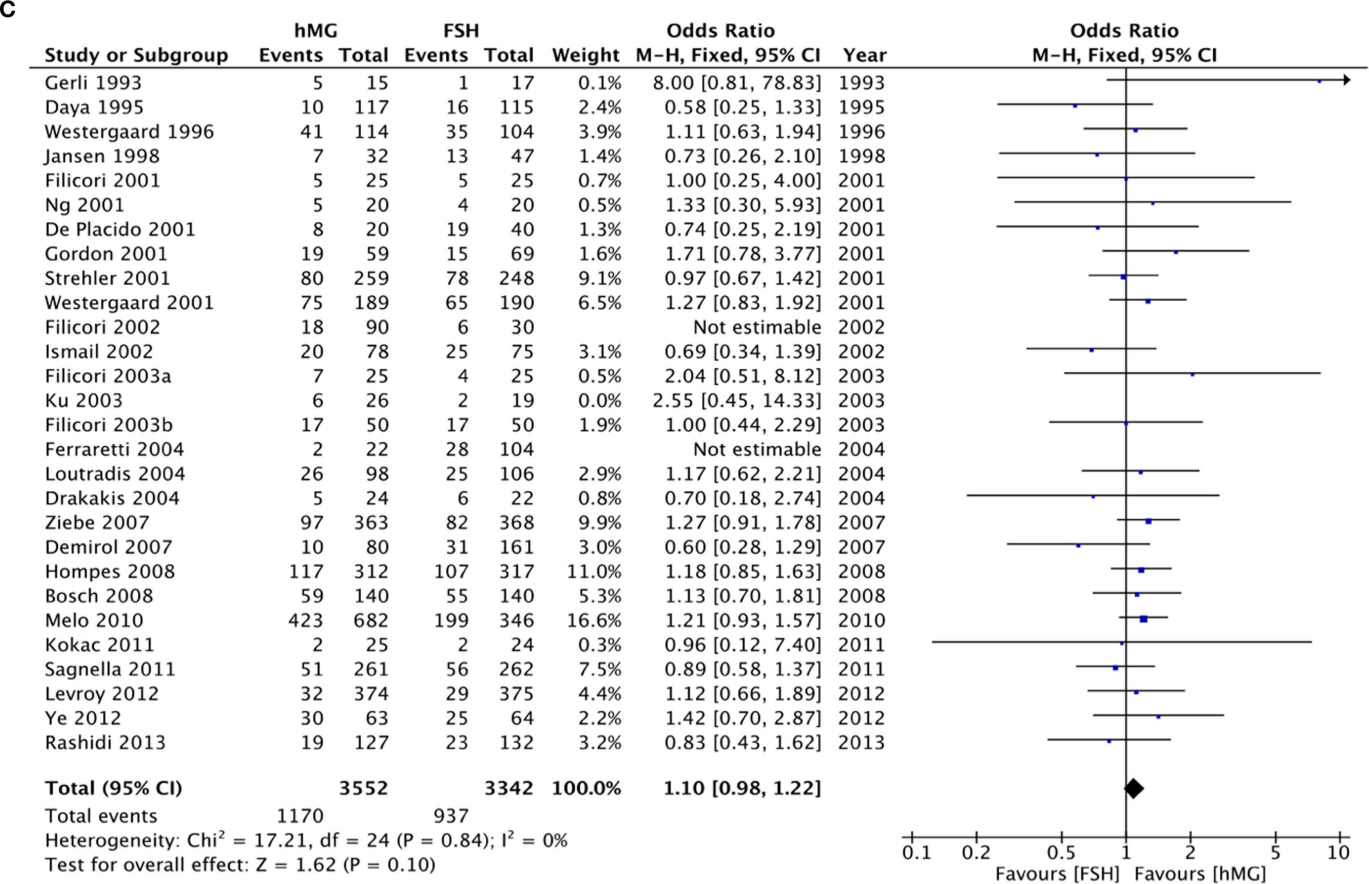
**Forrest plot evaluating the pregnancy rate comparing follicle-stimulating hormone alone to luteinizing hormone (A), human chorionic gonadotropin (B), and human menopausal gonadotropin (C)**.

The pregnancy rate was significantly higher when LH was added to FSH (*p* = 0.004), as evaluated in 29 studies for a total of 5,565 women enrolled (Figure 3A; Table 4).

Similarly, the higher pregnancy rate for the FSH plus LH group was maintained only when a GnRH agonist was used (*p* = 0.002), not with GnRH antagonist (*p* = 0.480) (Table 4).

Eight studies compared the use of FSH alone vs. FSH + hCG, for a total of 968 patients. The overall analysis did not find significant differences in pregnancy rate between groups (*p* = 0.750) (Figure 3B; Table 4).

Twenty-five studies compared hMG vs. FSH during COS, for a total of 6,894 patients. Pregnancy rate did not differ between groups (*p* = 0.100) (Figure 3C; Table 4). However, pregnancy rate was significantly higher when hMG was used in a GnRH agonist protocol (*p* = 0.030), while it did not change in a GnRH antagonist regimen (*p* = 0.370) (Table 4). In the comparison between hMG vs. FSH alone, considering only IVF/ICSI cycles, 22 studies remained in the analysis, for a total of 6,354 patients. Pregnancy rate did not differ between groups (*p* = 0.070) (Figure S1 in Supplementary Material). Considering only GnRH agonist protocols, 18 studies remained in the analysis, confirming the improved pregnancy rate in hMG group vs. FSH alone (*p* = 0.003) (Figure S2 in Supplementary Material).

Finally, five studies evaluated pregnancy rate comparing FSH + LH vs. FSH + hCG, for a total of 989 women. A higher pregnancy rate was observed when LH was added to FSH, rather than hCG (*p* < 0.001) (Table 4).

### Live Birth Rate

Five studies reported the live birth rate in the comparison of FSH alone vs. FSH + LH, without significant differences (*p* = 0.150) (Table 4). Similar result was obtained when FSH alone was compared to FSH + hCG (8 studies, *p* = 0.750) and to hMG (7 studies, *p* = 0.170) (Table 4).

### Meta-Regression Analyses

Considering each subgroup analysis, the number of oocytes retrieved was directly related to the cumulative FSH dose when FSH alone was used (*R* = 0.342, *p* = 0.002), instead of the combination FSH + LH (*R* = 0.146, *p* = 0.060). On the contrary, the cumulative FSH dose was not related to the oocytes number when FSH was compared to hMG (*R* = 0.022, *p* = 0.543).

### Risk-of-Bias

**Figure 4:**
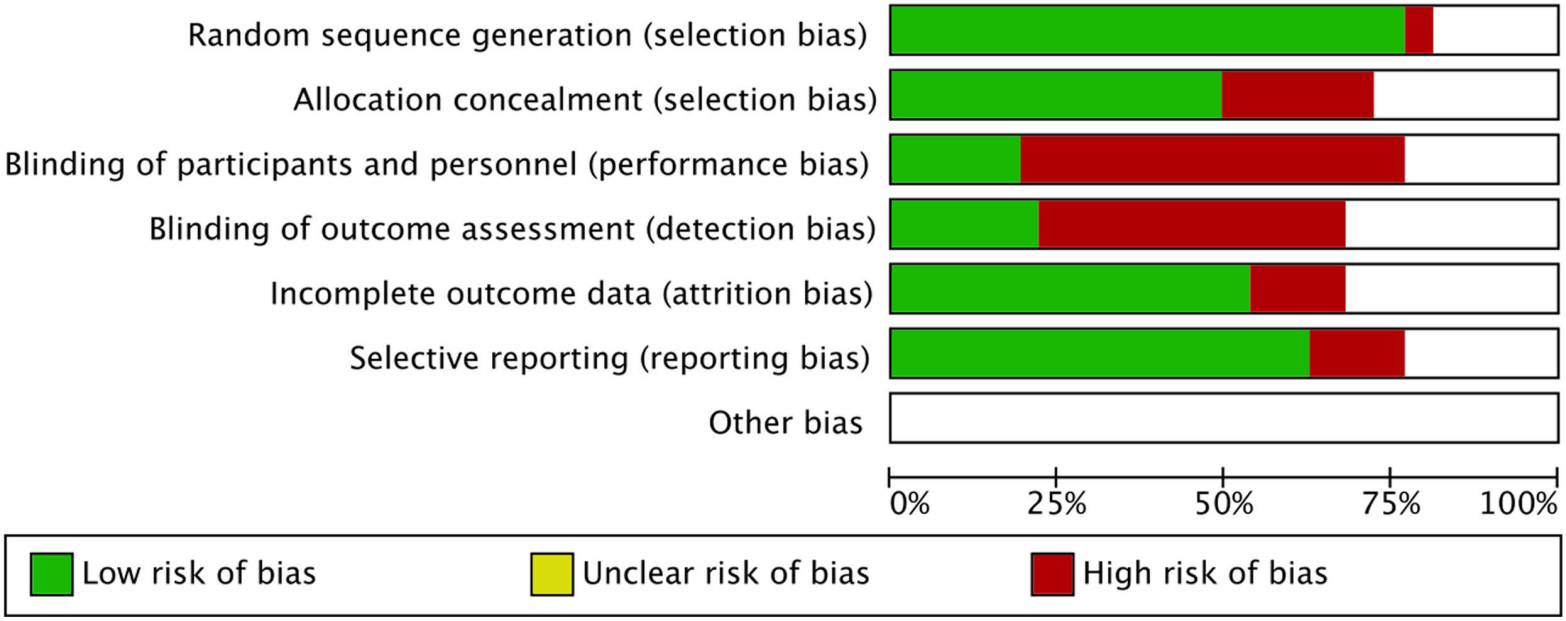
**Risk-of-bias graph: the authors’ judgment about each risk-of-bias item is presented as percentages across all included studies**.

The risk-of-bias was evaluated and summarized in Figure 4.

### Overall Model

**Figure 5:**
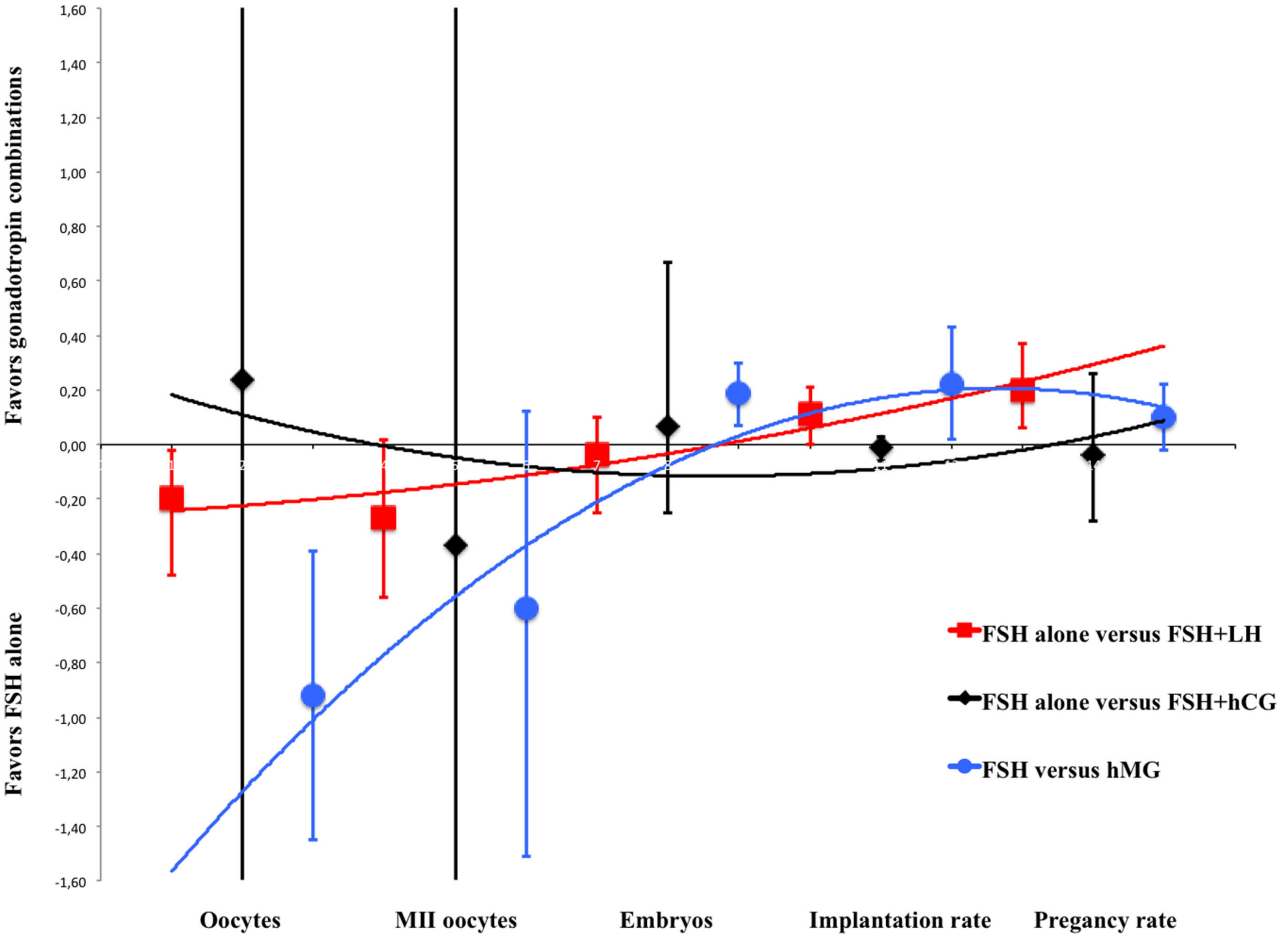
**Overall model of meta-analysis results**. Each scatter plot represents the mean differences with related confidence interval (95%) for each of assisted reproductive technology outcomes evaluated. The three lines represent the polynomial trend line. Red line shows the results with luteinizing hormone supplementation, blue line with human menopausal gonadotropin and black line with human chorionic gonadotropin.

The main concepts found by our data analysis were graphically summarized by a plot (Figure 5), representing the means and 95% confidence intervals of each fertilization step and gonadotropin regimen as extensively detailed in the subchapters above. In this overall model, COS served as an example of gonadotropins efficacy *in vivo* illustrating LH and hCG action on the ovary (Figure 5). Second-order polynomial functions were used as a fitting model of the standard mean differences (on the Y axis) calculated for each endpoint of the meta-analysis, considering FSH + LH vs. FSH alone, FSH + hCG vs. FSH alone and hMG vs. FSH (Figure 5). The number of oocytes retrieved is higher when FSH is used alone in all comparison, but the addition of LH or LH activity (such as in the case of hMG) progressively improves the ART outcomes, suggesting a positive effect of LH on oocyte quality. Especially, MII oocytes, embryos, implantation rate, and pregnancy rate improve progressively and linearly when LH is used (red line), an effect attenuated when hMG is used (blue line) (Figure 5). On the contrary, hCG addition does not improve ART outcome (black line) (Figure 5).

## Discussion

This is the first meta-analysis comparing comprehensively the efficacy of the mostly used gonadotropin combinations in ART. We find that the administration of FSH alone during COS retrieves higher oocyte number than either LH supplementation or hMG use. However, the combined use of FSH + LH reduces the FSH dose required for oocyte retrieved, while hMG leads to higher FSH dose needed. Interestingly, FSH + LH increases the pregnancy rate of about 1.20 fold, in spite of lower number of oocyte retrieved compared to FSH alone, whereas hMG does not. On the contrary, FSH + hCG treatment does neither change final oocytes number, nor FSH dose required for each oocyte, nor pregnancy rate. Although live birth rate is usually considered a better endpoint than pregnancy rate to evaluate ART outcome, it is not reported in many studies included and our meta-analysis does not show significant difference in live birth rate. All these differences are modest but, although apparently not clinically relevant, they are useful to better understand *in vivo* the overall effects of the different gonadotropin regimens.

These results suggest that gonadotropin preparations differently influence COS outcome, providing some evidence for ART personalization and improvement and leading to different results compared to those of previous meta-analyses. This difference could be due to the wide range of studies evaluated, which are focused on different endpoints and patient characteristics. FSH + LH treatment is linked to a relatively lower number of oocytes retrieved but higher pregnancy rate. The addition of LH or LH-activity might increase the selective pressure exerted on follicular selection exerted by the two gonadotropins together, compensated by improved oocyte quality. Indeed, the differences between FSH alone and FSH + LH or LH activity are lost, at least in terms of MII oocyte number. Moreover, the use of hMG leads to a higher embryos number and implantation rate compared to FSH alone. These results confirmed that the higher pregnancy rate found when FSH + LH or hMG are used together with GnRH agonist protocol, instead of FSH alone, is due to a positive effect of better oocyte quality on fertilization and embryo implantation. On the contrary, FSH + hCG treatment does not change ART outcomes compared to FSH alone, suggesting that LH and hCG result in different actions *in vivo* in the presence of FSH, reflecting *in vitro* observations (3). The overall model (Figure 5) shows a progressively better outcome when FSH is used together with LH or LH-activity (such as hMG). Thus, LH and hCG action *in vivo* is different in women undergoing COS, with LH improving oocyte maturation and quality, and therefore pregnancy rate, more than hCG, reflecting previous *in vitro* data.

Luteinizing hormone and hCG are characterized by specific molecular and biochemical features; they interact with distinct binding sites of the same receptor (25–27), resulting in lower dissociation rate by hCG than LH binding (28). Gonadotropin-specific ligand-receptor features imply different gene expression and intracellular signaling *in vitro*, whereby LH triggers higher levels of ERK1/2- and AKT-pathway activation than hCG, which, in turn, mediates more potent cAMP increase in human primary granulosa cells (2). Downstream effects of gonadotropins’ signaling consist in LH-related proliferative and anti-apoptotic signals, vs. high steroidogenic potential and pro-apoptotic activity of hCG *in vitro*, in both human and goat primary granulosa cells (3, 29). In particular, cell death was described as a result of the intracellular cross-talk among cAMP/protein kinase A (PKA)-mediated steroidogenic and pro-apoptotic pathways (30) preferentially activated by FSH and hCG, in steroidogenic cells *in vitro* (31).

Interestingly, our analysis of the literature reveals that LH addition to FSH treatment for ART provides lower oocyte numbers than other treatments, probably as a result of higher follicular selection (which is apoptosis-mediated). In this regard, few speculative considerations should be done. First, COS cycles are far from being a physiologic hormonal regimen; they are optimized for multi-follicular maturation in order to obtain the highest number of healthy oocytes (32), subjecting ovaries to treatments with pre-designed, high doses of exogenous hormones, which change the natural endocrine *milieu* of the woman. As a result, a mono-ovulatory species becomes multi-ovulatory, deviating from the natural, cyclic balance between gonadotropins and steroid hormones (33) and, thereby, life/death signals, a situation clearly different from ovarian physiology. On the other hand, FSH and LH are naturally produced to regulate mono-follicular selection, growth and maturation. The message provided by *in vitro* studies is that highly steroidogenic gonadotropins, i.e., FSH and hCG, mediate apoptotic stimuli in granulosa cells *via* cAMP/PKA-pathway (2, 3, 29–31). In the ovarian setting of a multi-follicular maturation as in COS, stimulation is a potent signal for early tertiary follicle recruitment (34) and triggering steroidogenesis, results in estrogen over-production which, in turn, induces more pronounced multi-follicular survival and maturation (35) than that inducible by LH treatment.

The ART outcome obtained with hMG reflects the heterogeneity typical of this compound. hMG derives from post-menopausal or pregnant women and contains both FSH and LH activities (36). LH activity is provided by residual LH molecules and by hCG supplementation, leading to high variability of the product (37). Moreover, given the high steroidogenic potential of hCG demonstrated *in vitro* (2, 31), which is more similar to that of FSH rather than LH (31), it is not surprising that ART outcome does not change whether hMG is used instead of FSH, except in GnRH agonist protocols, where high oocyte numbers might possibly occur as a positive effect of the flare-up phase on follicle recruitment. The discrepancy provided by GnRH-agonist and -antagonist protocols was not demonstrated by previous meta-analyses, likely due to strict inclusion criteria focused specifically on the evaluation of the analog instead of gonadotropins combination. The most recent meta-analysis on this field suggests only a significant adverse events occurrence reduction when GnRH antagonists are used (38).

This study suggests that GnRH antagonist protocol may be disadvantageous for oocytes quality, although the addition of LH seems to compensate, at least in part, this negative effect. FSH alone allows higher number of oocytes retrieved than FSH + LH, in GnRH agonist, but not antagonist protocols. GnRH antagonist is linked to lower FSH doses required for each oocyte retrieved, in the presence of LH. Moreover, pregnancy rate is higher by hMG than FSH treatment in GnRH agonist, but not antagonist protocols. This reflects the different mechanism of action and possibly different effects among GnRH analogs, which was hypothesized, although largely debated (39). GnRH analogs are differently used in clinical practice. In particular, GnRH agonists are generally proposed in women with BMI <25 kg/m^2^ (40), in poor responders (38, 41), and/or as a final trigger to minimize the ovarian hyperstimulation syndrome (OHSS) occurrence (42). Overall, GnRH antagonist is linked to reduced COS duration and overall medical costs of the stimulation phase and is recommended when a mild stimulation is required, such as for hyper-responder women (38, 43) or PCOS patients (44). These results support the hypothetical difference between agonists and antagonists, which was never demonstrated by previous meta-analyses (Table 1).

With this in mind, the cost-effectiveness evaluation currently remains the main variable useful to guide the clinician choice in the setting of the personalized therapy (45). However, the assessment of ART costs is particularly challenging, and the consideration of both COS-related and pregnancy/infant-associated medical costs is mandatory. Several studies evaluated the ART medical costs alone, considering the cumulative gonadotropin dosages used, the cycle cancelation rate and the risk of adverse events. The FASTT study suggested that IUI was the cheapest/efficient first-line treatment (46), while the FORT-T trial suggested better cost-effectiveness results when sequential traditional embryo transfer is selected (47). Crawford et al. (48) recently evaluated the overall ART costs in 14,398 cycles, suggesting that sequential embryo transfer is more expensive, concerning the procedure costs, but markedly cheaper overall, reducing multiple live births and total, final expenses. Although each study seems to be conclusive, these results remain challenging, and international or national consensus on the best COS approach is not reached so far. Moreover, the gonadotropin combination is not generally considered in this cost-effectiveness evaluation, limiting the strength of these suggestions. Our results suggest a reduced FSH dose needed for each oocyte retrieved when the combination of FSH + LH was used for COS. Thus, the gonadotropin combination should be considered in the cost-saving evaluation of a specific ART procedure. The overall charge, even when LH, hCG, or hMG are used in addition/substitution to FSH, must be considered according to the local reimbursement system. Finally, no study so far evaluated the “weight” of gonadotropin-producing companies on the clinician’s decision.

The main limit of this meta-analysis is the heterogeneity of studies included as suggested by the elevated I^2^ score. Couple infertility represents a challenging clinical condition, difficult to define according to strict clinical criteria. Indeed, different inclusion and exclusion criteria are used in each trial, making the comprehensive comparison of these results difficult. As a confirmation, a recent phase III single-blind, randomized, parallel-group clinical trial performed on 939 poor responder women did not find any safety and efficacy differences between FSH alone and FSH + LH (49). This reinforces the knowledge of a high heterogeneity of studies in ART setting, in which also the women classification as poor responders could mask the different gonadotropin effects *in vivo*. The relative high risk-of-bias of the studies included, as shown in Figure 4, represents an important limit that should be carefully considered to design further appropriate studies. However, although the pharmacological approach to ART is evaluated, no publication biases are evident at funnel plots analyses (data not shown). As highlighted by previous meta-analyses, we found high selection and allocation biases, confirming the finding that more than 80% of clinical trials did not apply any blinding technique (50). This high percentage is probably due to the difficulty in applying these procedures to ART, in which over 30 therapeutic complex approaches are currently available.

In conclusion, we found that different performance in ART is depending on gonadotropin combination used for COS, reflecting the physiological role of these molecules as previously indicated by *in vitro* data. This leads to important implication for clinical practice, where pregnancy rate or oocyte numbers might be the preferentially selected outcome. Especially, LH addition to FSH decreases FSH need and progressively improves ART outcomes and pregnancy rate. In GnRH agonist protocols, a better pregnancy rate is obtained by FSH + LH and hMG treatment. FSH + hCG or hMG alone are equally effective compared to FSH alone on pregnancy rate.

## Author Contributions

DS and LC searched and evaluated separately the studies. All authors participated to the analysis, discussion of the results, and manuscript preparation.

## Conflict of Interest Statement

The authors declare that there is no conflict of interest that could be perceived as prejudicing the impartiality of the research reported.

## Supplementary Material

Figure S1 **Forrest plot evaluating the pregnancy rate comparing follicle-stimulating hormone to human menopausal gonadotropin in *in vitro* fertilization/intracytoplasmic injection protocols alone**.

Figure S2 **Forrest plot evaluating the pregnancy rate comparing follicle-stimulating hormone to human menopausal gonadotropin in *in vitro* fertilization/intracytoplasmic injection protocols alone, using gonadotropin-releasing hormone agonist**.

Table S1 **PRISMA 2009 Checklist**.

Data Sheet 1 **Results**.

## References

[1] Ascoli M Fanelli F Segaloff DL. The lutropin/choriogonadotropin receptor, a 2002 perspective. Endocr Rev (2002) 23(2):141–74. 10.1210/edrv.23.2.0462 11943741

[2] Casarini L Lispi M Longobardi S Milosa F La Marca A Tagliasacchi D LH and hCG action on the same receptor results in quantitatively and qualitatively different intracellular signalling. PLoS One (2012) 7(10):e46682. 10.1371/journal.pone.0046682 23071612 PMC3465272

[3] Casarini L Riccetti L De Pascali F Nicoli A Tagliavini S Trenti T Follicle-stimulating hormone potentiates the steroidogenic activity of chorionic gonadotropin and the anti-apoptotic activity of luteinizing hormone in human granulosa-lutein cells in vitro. Mol Cell Endocrinol (2016) 422:103–14. 10.1016/j.mce.2015.12.008 26690776

[4] Baer G Loumaye E Comparison of recombinant human luteinising hormone (r-hLH) and human menopausal gonadotropin (hMG) in assisted reproductive technology. Curr Med Res Opin (2003) 19(2):83–8. 10.1185/030079902125001498 12740151

[5] Wong KM Mastenbroek S Repping S Cryopreservation of human embryos and its contribution to in vitro fertilization success rates. Fertil Steril (2014) 102(1):19–26. 10.1016/j.fertnstert.2014.05.027 24890275

[6] Alviggi C Humaidan P Ezcurra D. Hormonal, functional and genetic biomarkers in controlled ovarian stimulation: tools for matching patients and protocols. Reprod Biol Endocrinol (2012) 10:9. 10.1186/1477-7827-10-9 22309877 PMC3299595

[7] Davis OK. IVF stimulation: protocols for poor responders. Methods Mol Biol (2014) 1154:329–41. 10.1007/978-1-4939-0659-8_15 24782017

[8] Shrestha D La X Feng HL. Comparison of different stimulation protocols used in in vitro fertilization: a review. Ann Transl Med (2015) 3(10):137. 10.3978/j.issn.2305-5839.2015.04.09 26207230 PMC4486909

[9] Edwards RG Steptoe PC. Induction of follicular growth, ovulation and luteinization in the human ovary. J Reprod Fertil Suppl (1975) 22:121–63. 1058970

[10] Cedrin-Durnerin I Grange-Dujardin D Laffy A Parneix I Massin N Galey J Recombinant human LH supplementation during GnRH antagonist administration in IVF/ICSI cycles: a prospective randomized study. Hum Reprod (2004) 19(9):1979–84. 10.1093/humrep/deh369 15192072

[11] Propst AM Hill MJ Bates GW Palumbo M Van Horne AK Retzloff MG. Low-dose human chorionic gonadotropin may improve in vitro fertilization cycle outcomes in patients with low luteinizing hormone levels after gonadotropin-releasing hormone antagonist administration. Fertil Steril (2011) 96(4):898–904. 10.1016/j.fertnstert.2011.06.069 21839437

[12] Balasch J Fabregues F. LH in the follicular phase: neither too high nor too low. Reprod Biomed Online (2006) 12(4):406–15. 10.1016/S1472-6483(10)61991-8 16740211

[13] Hompes PG Broekmans FJ Hoozemans DA Schats R FIRM group. Effectiveness of highly purified human menopausal gonadotropin vs. recombinant follicle-stimulating hormone in first-cycle in vitro fertilization-intracytoplasmic sperm injection patients. Fertil Steril (2008) 89(6):1685–93. 10.1016/j.fertnstert.2007.05.039 17681325

[14] Jadad AR Moore RA Carroll D Jenkinson C Reynolds DJ Gavaghan DJ Assessing the quality of reports of randomized clinical trials: is blinding necessary? Control Clin Trials (1996) 17(1):1–12. 10.1016/0197-2456(95)00134-4 8721797

[15] Sunkara SK Rittenberg V Raine-Fenning N Bhattacharya S Zamora J Coomarasamy A. Association between the number of eggs and live birth in IVF treatment: an analysis of 400 135 treatment cycles. Hum Reprod (2011) 26(7):1768–74. 10.1093/humrep/der106 21558332

[16] Drakopoulos P Blockeel C Stoop D Camus M de Vos M Tournaye H Conventional ovarian stimulation and single embryo transfer for IVF/ICSI. How many oocytes do we need to maximize cumulative live birth rates after utilization of all fresh and frozen embryos? Hum Reprod (2016) 31(2):370–6. 10.1093/humrep/dev316 26724797

[17] Toftager M Bogstad J Løssl K Prætorius L Zedeler A Bryndorf T Cumulative live birth rates after one ART cycle including all subsequent frozen-thaw cycles in 1050 women: secondary outcome of an RCT comparing GnRH-antagonist and GnRH-agonist protocols. Hum Reprod (2017) 32(3):556–67. 10.1093/humrep/dew358 28130435

[18] Filicori M Cognigni GE Pocognoli P Tabarelli C Spettoli D Taraborrelli S Modulation of folliculogenesis and steroidogenesis in women by graded menotrophin administration. Hum Reprod (2002) 17(8):2009–15. 10.1093/humrep/17.8.2009 12151429

[19] Ku SY Suh CS Kim SH Choi YM Kim JG Moon SY. A pilot study of the use of low dose human menopausal gonadotropin in ovulation induction. Eur J Obstet Gynecol Reprod Biol (2003) 109(1):55–9. 10.1016/S0301-2115(02)00476-1 12818444

[20] Ferraretti AP Gianaroli L Magli MC D’Angelo A Farfalli V Montanaro N. Exogenous luteinizing hormone in controlled ovarian hyperstimulation for assisted reproduction techniques. Fertil Steril (2004) 82(6):1521–6. 10.1016/j.fertnstert.2004.06.041 15589853

[21] Kocak M Dilbaz B Demir B Tasci Y Tarcan A Dede S Lyophilised hMG versus rFSH in women with unexplained infertility undergoing a controlled ovarian stimulation with intrauterine insemination: a prospective, randomised study. Gynecol Endocrinol (2010) 26(6):429–34. 10.3109/09513591003632175 20175705

[22] Sagnella F Moro F Lanzone A Tropea A Martinez D Capalbo A A prospective randomized noninferiority study comparing recombinant FSH and highly purified menotropin in intrauterine insemination cycles in couples with unexplained infertility and/or mild-moderate male factor. Fertil Steril (2011) 95(2):689–94. 10.1016/j.fertnstert.2010.08.044 20869704

[23] Rashidi M Aaleyasin A Aghahosseini M Loloi S Kokab A Najmi Z. Advantages of recombinant follicle-stimulating hormone over human menopausal gonadotropin for ovarian stimulation in intrauterine insemination: a randomized clinical trial in unexplained infertility. Eur J Obstet Gynecol Reprod Biol (2013) 169(2):244–7. 10.1016/j.ejogrb.2013.03.002 23541417

[24] Moro F Scarinci E Palla C Romani F Familiari A Tropea A Highly purified hMG versus recombinant FSH plus recombinant LH in intrauterine insemination cycles in women >/=35 years: a RCT. Hum Reprod (2015) 30(1):179–85. 10.1093/humrep/deu302 25398971

[25] Muller T Gromoll J Simoni M Absence of exon 10 of the human luteinizing hormone (LH) receptor impairs LH, but not human chorionic gonadotropin action. J Clin Endocrinol Metab (2003) 88(5):2242–9. 10.1210/jc.2002-021946 12727981

[26] Grzesik P Teichmann A Furkert J Rutz C Wiesner B Kleinau G Differences between lutropin-mediated and choriogonadotropin-mediated receptor activation. FEBS J (2014) 281(5):1479–92. 10.1111/febs.12718 24438591

[27] Grzesik P Kreuchwig A Rutz C Furkert J Wiesner B Schuelein R Differences in signal activation by LH and hCG are mediated by the LH/CG receptor’s extracellular hinge region. Front Endocrinol (2015) 6:140. 10.3389/fendo.2015.00140 26441830 PMC4585211

[28] Huhtaniemi IT Catt KJ Differential binding affinities of rat testis luteinizing hormone (LH) receptors for human chorionic gonadotropin, human LH, and ovine LH. Endocrinology (1981) 108(5):1931–8. 10.1210/endo-108-5-1931 6260468

[29] Gupta C Chapekar T Chhabra Y Singh P Sinha S Luthra K Differential response to sustained stimulation by hCG & LH on goat ovarian granulosa cells. Indian J Med Res (2012) 135:331–40. 22561619 PMC3361869

[30] Amsterdam A Gold RS Hosokawa K Yoshida Y Sasson R Jung Y Crosstalk among multiple signaling pathways controlling ovarian cell death. Trends Endocrinol Metab (1999) 10(7):255–62. 10.1016/S1043-2760(99)00164-2 10461171

[31] Casarini L Reiter E Simoni M beta-arrestins regulate gonadotropin receptor-mediated cell proliferation and apoptosis by controlling different FSHR or LHCGR intracellular signaling in the hGL5 cell line. Mol Cell Endocrinol (2016) 437:11–21. 10.1016/j.mce.2016.08.005 27502035

[32] Zech NH Zech M Baldauf S Comploj G Murtinger M Spitzer D Ovarian stimulation in ART – unwinding pressing issues. Minerva Ginecol (2015) 67(2):127–47. 25668422

[33] Detti L Saed GM Fletcher NM Kruger ML Brossoit M Diamond MP. Endometrial morphology and modulation of hormone receptors during ovarian stimulation for assisted reproductive technology cycles. Fertil Steril (2011) 95(3):1037–41. 10.1016/j.fertnstert.2010.12.025 21227412 PMC3769103

[34] Dewailly D Robin G Peigne M Decanter C Pigny P Catteau-Jonard S Interactions between androgens, FSH, anti-Mullerian hormone and estradiol during folliculogenesis in the human normal and polycystic ovary. Hum Reprod Update (2016) 22:709–22. 10.1093/humupd/dmw027 27566840

[35] Ting AY Xu J Stouffer RL. Differential effects of estrogen and progesterone on development of primate secondary follicles in a steroid-depleted milieu in vitro. Hum Reprod (2015) 30(8):1907–17. 10.1093/humrep/dev119 26040480 PMC4507328

[36] Ezcurra D Humaidan P. A review of luteinising hormone and human chorionic gonadotropin when used in assisted reproductive technology. Reprod Biol Endocrinol (2014) 12:95. 10.1186/1477-7827-12-95 25280580 PMC4287577

[37] Lehert P Schertz JC Ezcurra D. Recombinant human follicle-stimulating hormone produces more oocytes with a lower total dose per cycle in assisted reproductive technologies compared with highly purified human menopausal gonadotrophin: a meta-analysis. Reprod Biol Endocrinol (2010) 8:112. 10.1186/1477-7827-8-112 20846363 PMC2954883

[38] Al-Inany HG Youssef MA Ayeleke RO Brown J Lam WS Broekmans FJ. Gonadotrophin-releasing hormone antagonists for assisted reproductive technology. Cochrane Database Syst Rev (2016) 4:CD001750. 10.1002/14651858.CD001750.pub4 27126581 PMC8626739

[39] Orvieto R Patrizio P. GnRH agonist versus GnRH antagonist in ovarian stimulation: an ongoing debate. Reprod Biomed Online (2013) 26(1):4–8. 10.1016/j.rbmo.2012.11.001 23186555

[40] Rabinson J Meltcer S Zohav E Gemer O Anteby EY Orvieto R. GnRH agonist versus GnRH antagonist in ovarian stimulation: the influence of body mass index on in vitro fertilization outcome. Fertil Steril (2008) 89(2):472–4. 10.1016/j.fertnstert.2007.03.007 17582402

[41] Berkkanoglu M Ozgur K. What is the optimum maximal gonadotropin dosage used in microdose flare-up cycles in poor responders? Fertil Steril (2010) 94(2):662–5. 10.1016/j.fertnstert.2009.03.027 19368912

[42] Alama P Bellver J Vidal C Giles J. GnRH analogues in the prevention of ovarian hyperstimulation syndrome. Int J Endocrinol Metab (2013) 11(2):107–16. 10.5812/ijem.5034 23825982 PMC3693668

[43] Fauser BC Devroey P. Why is the clinical acceptance of gonadotropin-releasing hormone antagonist cotreatment during ovarian hyperstimulation for in vitro fertilization so slow? Fertil Steril (2005) 83(6):1607–11. 10.1016/j.fertnstert.2005.02.011 15950626

[44] Gianaroli L Racowsky C Geraedts J Cedars M Makrigiannakis A Lobo RA. Best practices of ASRM and ESHRE: a journey through reproductive medicine. Fertil Steril (2012) 98(6):1380–94. 10.1016/j.fertnstert.2012.07.1164 23102857

[45] La Marca A Sunkara SK. Individualization of controlled ovarian stimulation in IVF using ovarian reserve markers: from theory to practice. Hum Reprod Update (2014) 20(1):124–40. 10.1093/humupd/dmt037 24077980

[46] Reindollar RH Regan MM Neumann PJ Levine BS Thornton KL Alper MM A randomized clinical trial to evaluate optimal treatment for unexplained infertility: the fast track and standard treatment (FASTT) trial. Fertil Steril (2010) 94(3):888–99. 10.1016/j.fertnstert.2009.04.022 19531445

[47] Goldman MB Thornton KL Ryley D Alper MM Fung JL Hornstein MD A randomized clinical trial to determine optimal infertility treatment in older couples: the Forty and Over Treatment Trial (FORT-T). Fertil Steril (2014) 101(6):e1571–2. 10.1016/j.fertnstert.2014.03.012 24796764 PMC4106674

[48] Crawford S Boulet SL Mneimneh AS Perkins KM Jamieson DJ Zhang Y Costs of achieving live birth from assisted reproductive technology: a comparison of sequential single and double embryo transfer approaches. Fertil Steril (2016) 105(2):444–50. 10.1016/j.fertnstert.2015.10.032 26604068 PMC5125029

[49] Humaidan P Chin W Rogoff D D’Hooghe T Longobardi S Hubbard J Efficacy and safety of follitropin alfa/lutropin alfa in ART: a randomized controlled trial in poor ovarian responders. Hum Reprod (2017) 32(3):544–55. 10.1093/humrep/dew360 28137754 PMC5850777

[50] Papathanasiou A Searle BJ King NM Bhattacharya S. Trends in ’poor responder’ research: lessons learned from RCTs in assisted conception. Hum Reprod Update (2016) 22(3). 10.1093/humupd/dmw001 26843539

