# Supplementary figures and images for: Efficacy of Follicle-Stimulating Hormone (FSH) Alone, FSH + Luteinizing Hormone, Human Menopausal Gonadotropin or FSH + Human Chorionic Gonadotropin on Assisted Reproductive Technology Outcomes in the “Personalized” Medicine Era: A Meta-analysis
Source: Front Endocrinol (Lausanne). 2017 Jun 1;8:114. doi: 10.3389/fendo.2017.00114 (PMC5451514; doi:10.3389/fendo.2017.00114)

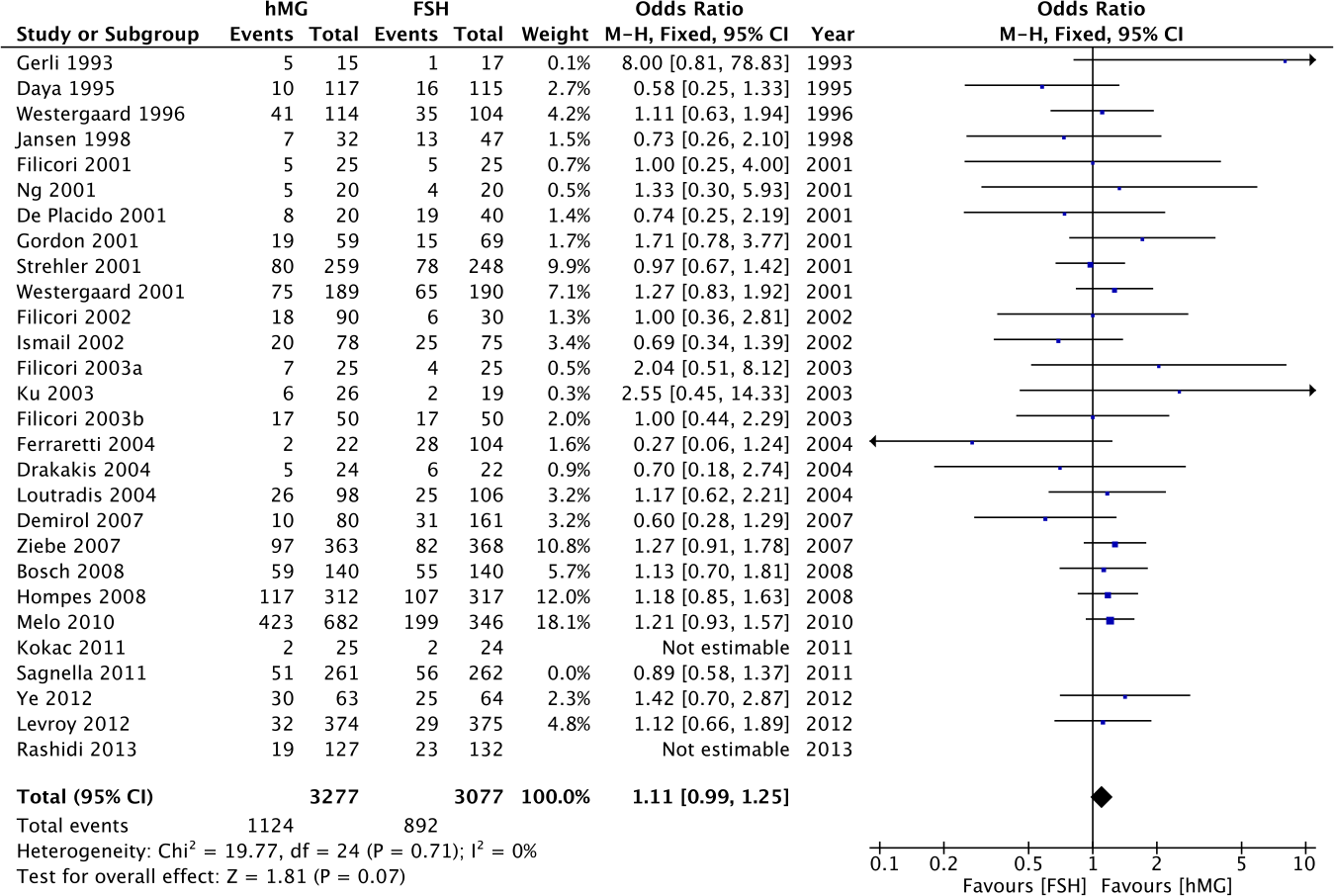

Supplement: Figure S1 — Forrest plot evaluating the pregnancy rate comparing follicle-stimulating hormone to human menopausal gonadotropin in in vitro fertilization/intracytoplasmic injection protocols alone. [file Image_1.TIFF]

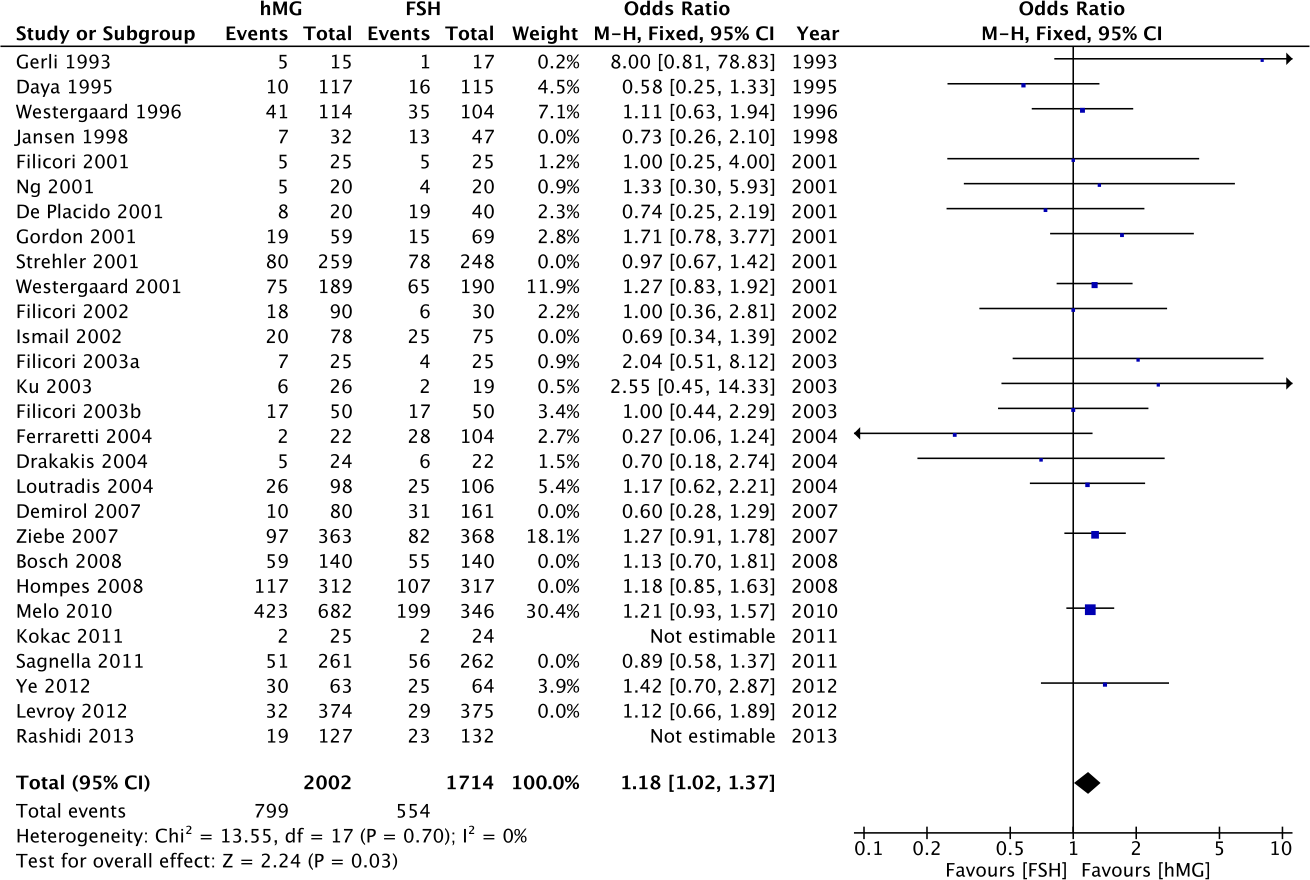

Supplement: Figure S2 — Forrest plot evaluating the pregnancy rate comparing follicle-stimulating hormone to human menopausal gonadotropin in in vitro fertilization/intracytoplasmic injection protocols alone, using gonadotropin-releasing hormone agonist. [file Image_2.TIFF]
