# Supplementary material for: Efficacy of Follicle-Stimulating Hormone (FSH) Alone, FSH + Luteinizing Hormone, Human Menopausal Gonadotropin or FSH + Human Chorionic Gonadotropin on Assisted Reproductive Technology Outcomes in the “Personalized” Medicine Era: A Meta-analysis
Source: Front Endocrinol (Lausanne). 2017 Jun 1;8:114. doi: 10.3389/fendo.2017.00114 (PMC5451514; doi:10.3389/fendo.2017.00114)
Supplement: Data Sheet 1 — Results. [file Data_Sheet_1.DOC]

**Results**

*Considerations on study design*

Since the mean age of patients was exceeding 35 years only in one fourth of the studies, the first subgroup analysis was performed by dividing the studies in two groups using the median age (32.5 years). Four studies were excluded from this subgroup analysis due to missing age reporting. Thirty-five studies enrolled women younger than 32.5 years (mean age 30.63+2.16 years), whereas 36 studies enrolled older women (mean age 35.72+2.44 years).

*Number of oocytes retrieved*

Studies using FSH alone retrieved a significantly higher number of oocytes compared to FSH + LH treatment when studies were sub-grouped for age above (p=0.030), but not below (p=0.500) the median age of 32.5 years.

The comparison FSH alone *versus* FSH + hCG did not find significant differences in the number of oocytes retrieved between groups both for studies under (p=0.510) and above the median age (p=0.820).

Number of oocytes retrieved was significantly higher in FSH than hMG group considering studies enrolling women younger than 32.5 years (p=0.020), but not older than 32.5 years (p=0.090).

In subgroup of studies omparing FSH plus LH to FSH plus hCG, the use of the age cut-off did not give more information, since only one study evaluated women younger than 32.5 years.

*FSH dose/retrieved oocyte ratio*

The FSH/retrieved oocyte ratio was significantly lower when LH was added to FSH when studies were sub-grouped for age below (p=0.020) and above 32.5 years (p<0.001).

The ratio between FSH dose and the number of oocytes retrieved was significantly lower in the FSH compared to hMG group considering both studies enrolling women younger than 32.5 years (p<0.001) and older than 32.5 years (p=0.002).

*Pregnancy rate*

The pregnancy rate was significantly higher when LH was added to FSH (for studies with age higher than 32.5 years (p=0.007). On the contrary, no significant differences in pregnancy rate were observed for studies enrolling women younger than 32.5 years (p=0.230).

Pregnancy rate remained not significantly different considering both studies enrolling women younger than 32.5 years (p=0.180) and older than 32.5 years (p=0.830), in the comparison between FSH alone and hMG. Moreover, subgroup analyses were performed, considering the age and the GnRH regimen together. The odds ratio of pregnancy rate did not increase when hMG was used in GnRH agonists protocols in younger women (1.05, CI: 0.85-1.30, p=0.640, I^2^=23%), whereas only in one study the GnRH agonist protocol was used in women older than 32.5 years. The use of GnRH antagonist did not lead to increase pregnancy rate, neither in young (1.06, CI: 0.79 -1.43, p=0.690, I^2^=1%) nor in older women (1.07, CI: 0.85 -1.35, p=0.550, I^2^=0%).
